# Novel tricyclic pyrazole BRAF inhibitors with imidazole or furan central scaffolds

**DOI:** 10.1016/j.bmc.2010.06.031

**Published:** 2010-09-15

**Authors:** Dan Niculescu-Duvaz, Ion Niculescu-Duvaz, Bart M.J.M. Suijkerbuijk, Delphine Ménard, Alfonso Zambon, Arnaud Nourry, Lawrence Davies, Helen A. Manne, Frank Friedlos, Lesley Ogilvie, Douglas Hedley, Andrew K. Takle, David M. Wilson, Jean-Francois Pons, Tom Coulter, Ruth Kirk, Neus Cantarino, Steven Whittaker, Richard Marais, Caroline J. Springer

**Affiliations:** a Cancer Research UK Centre for Cancer Therapeutics, The Institute of Cancer Research, 15 Cotswold Road, Sutton, Surrey SM2 5NG, United Kingdom; b The Institute of Cancer Research, Cancer Research UK Centre for Cell and Molecular Biology, 237 Fulham Road, London SW3 6JB, United Kingdom; c Department of Medicinal Chemistry, Neurology and GI Centre of Excellence for Drug Discovery, GlaxoSmithKline Pharmaceuticals, New Frontiers Science Park, Third Avenue, Harlow, Essex CM19 5AW, United Kingdom; d Evotec (UK) Ltd, 114 Milton Park, Abingdon, Oxfordshire OX14 4SA, United Kingdom

**Keywords:** Boc, *tert*-butoxycarbonyl, BRAF, V-RAF murine sarcoma viral oncogene homolog B1, DCM, dichloromethane, DMF, dimethylformamide, ERK, extracellular regulated kinase, MAPK, mitogen-activated protein kinase, MEK, MAPK/ERK kinase, MOM, methoxymethyl, PK, pharmacokinetics, RAF, rapidly growing fibrosarcoma, SAR, structure–activity relationship, TFA, trifluoroacetic acid, THF, tetrahydrofuran, BRAF, Kinase inhibitors, Anticancer, Melanoma, Triarylimidazole, Dihydroindenopyrazole

## Abstract

V-RAF murine sarcoma viral oncogene homolog B1 (BRAF) is a serine/threonine-specific protein kinase that is mutated with high frequency in cutaneous melanoma, and many other cancers. Inhibition of mutant BRAF is an attractive therapeutic approach for the treatment of melanoma. A triarylimidazole BRAF inhibitor bearing a phenylpyrazole group (dimethyl-[2-(4-{5-[4-(1*H*-pyrazol-3-yl)-phenyl]-4-pyridin-4-yl-1*H*-imidazol-2-yl}-phenoxy)-ethyl]-amine, **1a**) was identified as an active BRAF inhibitor. Based on this starting point, we synthesized a series of analogues leading to the discovery of 6-{2-[4-(4-methyl-piperazin-1-yl)-phenyl]-5-pyridin-4-yl-3*H*-imidazol-4-yl}-2,4-dihydro-indeno[1,2-*c*]pyrazole (**1j**), with nanomolar activity in three assays: inhibition of purified mutant BRAF activity in vitro; inhibition of oncogenic BRAF-driven extracellular regulated kinase (ERK) activation in BRAF mutant melanoma cell lines; and inhibition of proliferation in these cells.

## Graphical abstract

**1j** IC_50_ (BRAF) = 0.24 μM; IC_50_ (pERK) = 0.58 μM; GI_50_ (SRB) = 0.87 μM.

## 1 Introduction

The RAS-RAF-MEK-ERK pathway is hyper-activated in approximately 30% of human cancers,^1^ where it stimulates cell growth and survival. This hyper-activation is caused in part by mutations in receptor tyrosine kinases, the small G-proteins of the RAS family, and the serine/threonine specific protein kinase BRAF. Mutations in *BRAF* (one of the three rapidly growing fibrosarcoma (*RAF*) genes in humans; the other two being *ARAF* and *CRAF*) occur in ∼2% of human cancers, and are particularly prevalent in cutaneous melanomas (∼50% of cases). The most common mutation (90% frequency) is a glutamic acid for valine substitution at position 600 (V600E).^2^ In cancer cells, ^V600E^BRAF stimulates constitutive ERK activity and drives proliferation and survival, thereby providing essential tumor growth and maintenance functions.^3^ ^V600E^BRAF also contributes to neoangiogenesis by stimulating vascular endothelial growth factor secretion.^4^ Consequently, BRAF is an attractive validated therapeutic target in melanoma, and several BRAF inhibitors are currently under development and in clinical trials^5^ and we have previously described type II BRAF inhibitors, targeting the inactive conformation of BRAF.^6,7^

**Figure 1:**
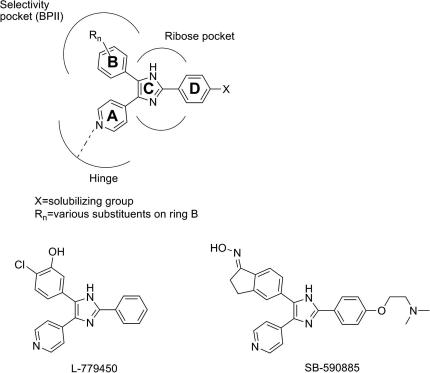
Examples of triarylimidazole BRAF inhibitors and their putative binding mode.

Triarylimidazoles have been reported as inhibitor scaffolds for targeting the active kinase conformation kinase, for example p38^8^ and RAF.^9,10^ The general structure of these latter kinase inhibitors contains a hinge-binding group, usually pyridine or pyrimidine (‘ring A’), a central imidazole scaffold occupying the ribose position in the ATP-binding pocket (‘ring C’) and a substituted aromatic group interacting with the hydrophobic pocket (BPII according to Liao’s nomenclature)^11^ next to the gatekeeper residue (‘ring B’) (Fig 1). The third aryl ring (‘ring D’) extends outside the ATP pocket towards the solvent. The co-crystal structure of BRAF with SB590885, a triarylimidazole BRAF inhibitor, indicates binding to the active conformation of BRAF and supports the described binding mode.^9^ Therefore, these compounds are BRAF type I inhibitors. Two examples of reported active triarylimidazole BRAF inhibitors, L-779450 and SB-590885, are also shown in Figure 1.^12,9^ These BRAF inhibitors contain an H-bond donor on ‘ring B’ (phenol or oxime, Fig. 1) that is essential for activity.

**Figure 2:**
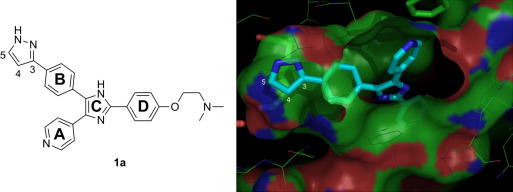
Structure of phenylpyrazole hit **1a,** and docking in BRAF structure.

In order fully to exploit the BPII pocket of the active BRAF conformation, we designed novel type I BRAF inhibitors based on this scaffold, but with an H-bond donor pyrazole heterocycle substituent on ‘ring B’, as a potential functional bioisostere of indanoneoxime and chlorophenol. 4-(3-Pyrazolyl)phenyl ring B was proposed to target the BPII pocket of BRAF and consequently compound **1a** was synthesized (Fig. 2). Docking of this compound with the BRAF structure in the active conformation (pdb code 2FB8) showed the expected orientation of the compound, similar to SB590885, with the pyridyl ring A interacting with the hinge and the 4-(3-pyrazolyl)phenyl ring B occupying the BPII pocket (Fig. 2). The geometry of the oxime in indanoneoxime inhibitors is dependent of the rest of the inhibitor: the co-crystal structure of BRAF with an indanoneoxime BRAF inhibitor with a pyrazole scaffold shows the oxime in *E*-geometry (pdb code 3D4Q),^13^ whereas SB590885 co-crystallises in an opposite *Z*-orientation. Pleasingly, this compound was found to inhibit isolated full length ^V600E^BRAF at low micromolar concentrations (IC_50_ BRAF = 1.6 μM) and also to inhibit ^V600E^BRAF signalling in melanoma cells (GI_50_ SRB = 7.4 μM). Hit **1a** was therefore used as starting point for the structure–activity relationship (SAR) exploration and structure optimization for new BRAF inhibitors.

## 2 Results and discussion

### 2.1 Chemistry

**Scheme 1:**
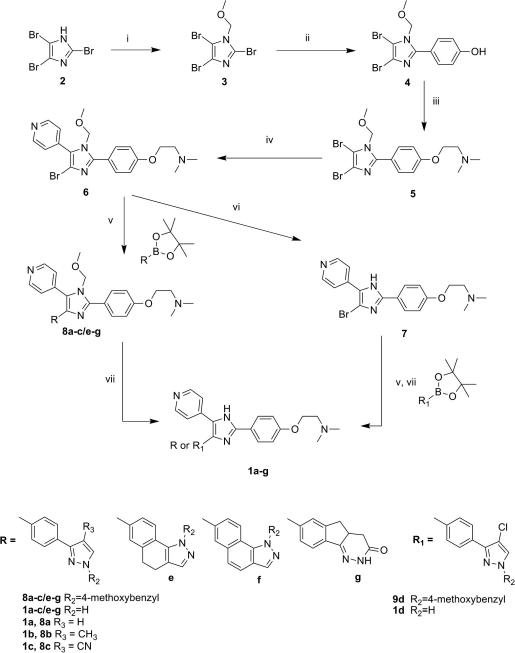
Synthesis of triarylimidazole pyrazoles by route A. Reagents and conditions: (i) MOMBr, NaH, THF; (ii) 4-HOC_6_H_4_B(OH)_2_, K_2_CO_3_, Pd(PPh_3_)_4_, toluene, MeOH, reflux; (iii) Cl(CH_2_)_2_N(CH_3_)_2_, Cs_2_CO_3_, DMF, 40 °C; (iv) pyridyl boronic ester, K_2_CO_3_, Pd(OAc)_2_, PPh_3_, DME, H_2_O, 90 °C; (v) boronic esters, K_2_CO_3_, Pd(OAC)_2_, PPh_3_, DME, H_2_O, 110 °C; (vi) 5 M HCl, 60 °C; (vii) TFA, 130 °C.

**Scheme 2:**
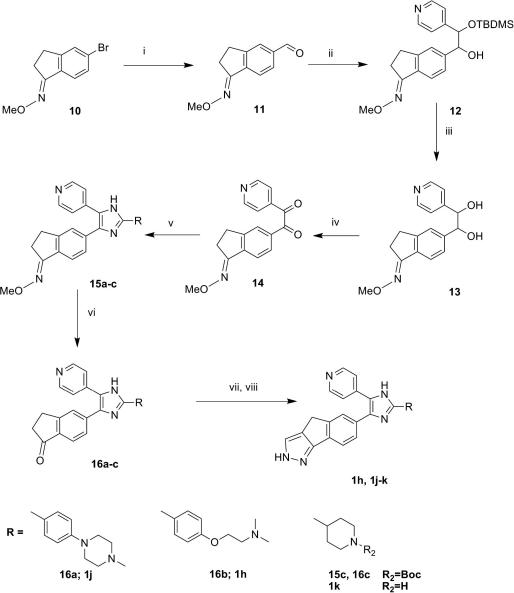
Synthesis of triarylimidazole tricyclic pyrazoles by route B. Reagents: (i) *n*BuLi, THF, DMF; (ii) 4-pyridyl-CH_2_OTBDMS, LDA, THF; (iii) TBAF, THF; (iv) oxalyl chloride, DMSO, Et_3_N, DMF; (v) RCHO, AcOH, AcONH_4_; (vi) 5 M HCl, dioxane; (vii) HC(NMe_2_)_3_, DMF; (viii) AcOH, H_2_NNH_2_.

Two synthetic routes were designed to access the desired trisubstituted imidazoles:

(A) Starting from the 2,4,5-tribromo-imidazole core, **2**, and introducing the desired substituents in sequence (Scheme 1).
(B) Using the protected indanone oxime (ring B) and building the trisubstituted imidazole core (Scheme 2).

In the first approach, the 2,4,5-tribromo-imidazole was protected as a methoxymethyl (MOM) derivative **3**, and converted using a two-step procedure to the 2-(4-dimethylaminoethoxyphenyl)-4,5-dibromoimidazole, **5**. The Suzuki coupling of this intermediate with 1 equiv of 4-pyridylboronic acid led to the key intermediate **6**. A second Suzuki coupling with 4-methoxybenzyl-protected boronic acids **26, 29a**–**b, 37, 40** or **43** (see Schemes 5 and 6) followed by acidic deprotection of the MOM and 4-methoxybenzyl groups generated the final compounds **1a**–**c**, **1e**–**g**. MOM deprotection of intermediate **6** led to imidazole **7**, which could be coupled directly with boronic acid **33,** to afford compound **1d** after 4-methoxybenzyl removal (Scheme 1).

The alternative route (B) is a modification of the synthesis reported by Takle et al.^10^ The starting material 5-bromo-2,3-dihydro-1*H*-inden-1-one *O*-methyl oxime **10**, was converted to the corresponding aldehyde **11** with *n*BuLi and (dimethylformamide) DMF, followed by coupling with 4-pyridylmethyleneoxy-*t*-butyldimethylsilane to afford intermediate **12**. After deprotection with tetrabutylammonium fluoride, the corresponding diol, **13**, was submitted to a Swern oxidation affording the dione **14**. The coupling of **14** with selected aldehydes afforded the imidazole core linked to a suitable ring D. The final deprotection of the *N*-methyl indanone oxime system yielded the ketones **16a**–**c**, which were converted in the final tricyclic compounds **1h, 1j**–**k** by condensation with tris-dimethylamino-methane and hydrazine (see Scheme 2).

**Scheme 3:**
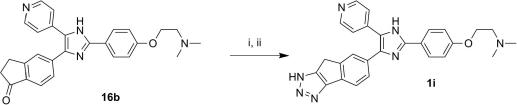
Synthesis of tricyclic triazole. Reagents and conditions: (i) *t*BuONO, HCl, methoxyethanol; (ii) ethylene glycol, KOH, H_2_NNH_2_, 190 °C.

The triazole final compound **1i** was obtained from intermediate **16b** by nitrosation followed by the addition of hydrazine (Scheme 3).

**Scheme 4:**
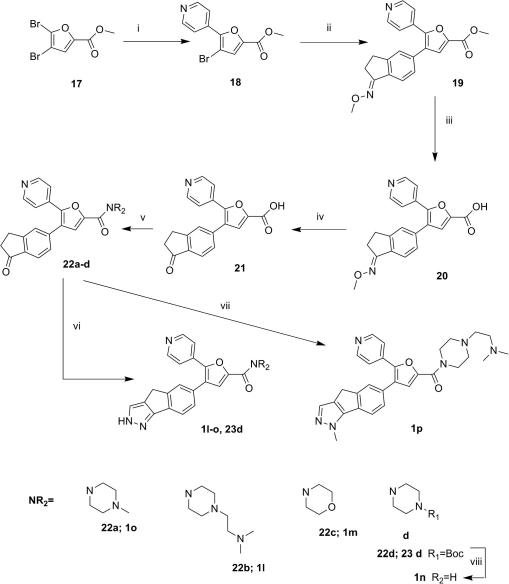
Synthesis of diarylfuranamides. Reagents: (i) 4-pyridylboronic acid, Cs_2_CO_3_, Ph_3_As, Pd(PPh_3_)Cl_2_, DMF; (ii) 1-(methoxyimino)-2,3-dihydro-1*H*-inden-5-ylboronic acid, Cs_2_CO_3_, Ph_3_As, Pd(PPh_3_)_2_Cl_2_, DMF; (iii) NaOH, THF, MeOH; (iv) 5 M HCl, dioxane–Me_2_CO; (v) amine, DIC, HOBt, TEA, DMF; (vi) DMF–DMA, H_2_NNH_2_, AcOH; (vii) DMF–DMA, MeHNNH_2_, AcOH; (viii) 4 N HCl.

Diarylfurans with aliphatic, aromatic or carboxamide solubilising groups were previously shown to be alternative scaffolds for the synthesis of BRAF inhibitors.^14^ Consequently, we synthesized several furan analogues with our most active tricyclic pyrazole ring B. For the synthesis of the trisubstituted furan analogues, the starting material was methyl-2,3-dibromofuran-5-yl carboxylate **17**. Using two consecutive Suzuki couplings with 4-pyridyl boronic acid followed by 1-(methoxyimino)-2,3-dihydro-1*H*-inden-5-ylboronic acid, the intermediate **19** was obtained. Basic hydrolysis of intermediate **19** with NaOH afforded the corresponding carboxylic acid, **20**, followed by acidic hydrolysis to remove the oxime ether group and generate the ketone **21**. Condensation with the desired amines, in the presence of diisopropylcarbodiimide, hydroxybenzotriazole and triethylamine in DMF, produced the key intermediates **22a**–**d**. The condensation of **22a**–**d** with hydrazine or methylhydrazine gave the desired final compounds **1l**–**o** and **1p**, respectively (Scheme 4).

**Scheme 5:**
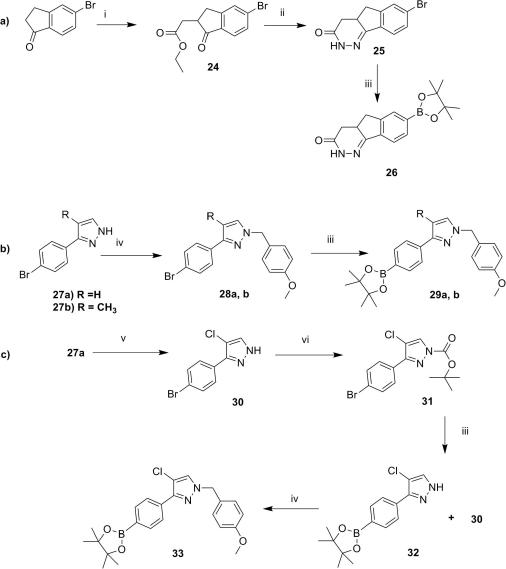
Synthesis of boronic acids **26, 29a, 29b** and **33**. Reagents and conditions: (i) 2M LDA, THF, BrCH_2_CO_2_Et; (ii) H_2_NNH_2_, EtOH; (iii) bis pinacolato-diboron, KOAc, Pd(dppf)Cl_2_, DMF, 90 °C; (iv) 4-methoxy benzyl chloride, NaH, DMF; (v) NCS, DCM; (vi) (BOC)_2_O, DMAP, CH_3_CN.

**Scheme 6:**
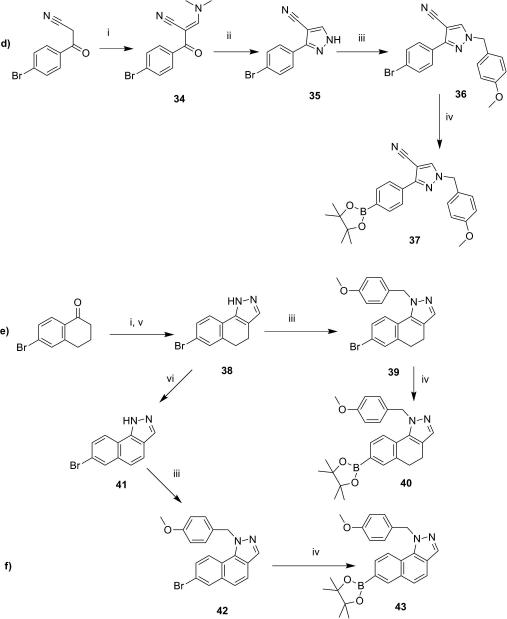
Synthesis of boronic acids **37, 40** and **43**. Reagents and conditions: (i) HC(NMe_2_)_3_, DMF; (ii) H_2_NNN_2_·H_2_O, EtOH; (iii) 4-methoxy benzyl chloride, NaH, DMF; (iv) bis pinacolato-diboron, KOAc, Pd(dppf)Cl_2_, DMF, 90 °C; (v) AcOH, H_2_NNH_2_; (vi) DDQ, dioxane.

Since most of the boronic acids used in the synthesis of the imidazoles previously described in Scheme 1 are not commercially available, their preparation is summarized in Schemes 5 and 6.

### 2.2 SAR of ring B—phenylpyrazole

In order to identify new type I BRAF inhibitors with a triarylimidazole scaffold, compound **1a** with 4-(3-pyrazolyl)phenyl as ring B was synthesized and found to be effective. The SAR of ring B was explored further, leading to the discovery of the more potent 1,4-dihydroindeno[1,2-*c*]pyrazole. Using this group, further SAR exploration of rings C and D was performed.

The biological activities of compounds **1a**–**p** were assessed in three assays:

(1) Inhibition of the isolated ^V600E^BRAF kinase activity (IC_50_ BRAF), measuring MEK1/2 phosphorylation as the endpoint.^15^ An alternative BRAF fluorescent ligand binding assay^10,14^ gave much lower *K*_d_ values than the IC_50_s for the ^V600E^BRAF kinase activity (for example: compound **1h,** *K*_d_ = 6.1 nM whereas IC_50_ BRAF = 230 nM). We measured the IC_50_ BRAF values in this paper, since they represent the inhibition of BRAF function which we consider more meaningful than the binding of inhibitor to BRAF.
(2) Inhibition of ^V600E^BRAF-dependent ERK phosphorylation in WM266.4 melanoma cells (IC_50_ pERK).
(3) Growth inhibition of WM266.4 melanoma cells measured by sulforhodamine-B (GI_50_ SRB). The IC_50_ (BRAF), IC_50_ (pERK) and GI_50_ (SRB) results are presented in Table 1.

Our assessment of substitution of the pyrazole ring focused on the 4-position, since no further space is available for position 5 based on modelling studies of **1a** with the BRAF structure reported in the co-crystal structure with SB590885 (Fig. 2).^9^ Substitution in the 4-position of the pyrazole with methyl (compound **1b**) is tolerated and beneficial. Larger substituents such as cyano or chlorine lead to loss of activity against both isolated BRAF and in cellular assays (**1c**, **1d**). This effect could be due to a steric clash with the BRAF binding pocket or an unfavourable electrostatic interaction. In order to fix the position of the pyrazole ring, more rigid tricyclic systems were synthesized: **1e**–**h**.

The five-membered ring (1,4-dihydroindeno[1,2-*c*]pyrazole **1h**) increases the potency against BRAF fivefold compared to the non-cyclic analogue **1b**. This tricyclic system mimics the indanoneoxime moiety, with the oxime group in the E-geometry. Saturated or aromatic six-membered rings (**1e**, **1f**) are not as effective as the five-membered ring. Methylation of the pyrazole leads to a loss in activity (**1p** vs **1l**) consistent with the hypothesis that an H-donor group is beneficial for potent BRAF inhibition. Interestingly, the tricyclic triazole analogue **1i** is inactive, possibly due to a polarity mismatch between the polar 3-position of the triazole (corresponding to the 5-position of the pyrazole) and a non-polar area of the BPII binding pocket. Similar results were observed with the tricyclic pyridazinone **1g**, where the inactivity can be attributed to either polarity mismatch or steric clash. Since the 1,4-dihydroindeno[1,2-*c*]pyrazole **1h** is the most active ring B, further optimization of rings C and D were performed keeping this ring B constant.

### 2.3 SAR of rings C and D

Furan-based analogues of SB-590885, where the core imidazole ring C was replaced by furan, with a variety of solubilizing D groups have been reported as potent BRAF inhibitors.^14^ We have synthesized a number of furan-amides with our tricyclic pyrazole ‘ring B’ as BRAF inhibitors (**1l**–**p**), which were found to possess weaker activity against BRAF than their imidazole counterparts, but surprisingly good cellular activity (for example compound **1m** has submicromolar activity in both IC_50_ ppERK and GI_50_ SRB assays).

Modification of the substituents on the phenyl ‘group D’ (solubilizing group) of the imidazole inhibitors is expected to have a minimal effect on the BRAF potency but could modulate the cellular activity or the physicochemical properties of this series. Indeed, the replacement of the 2-dimethylaminoethoxy side chain of ring D (in **1h**) with *N*-methylpiperazine lead to compound **1j**, which is equipotent on BRAF IC_50_ with **1h**, but has improved cellular activity. Compound **1j** has submicromolar activity in all 3 assays. Replacing the whole aryl ring D with a piperidine ring (**1k**) has a negative effect on BRAF potency, with a sixfold potency loss compared to **1h** or **1j**.

### 2.4 Pharmacokinetic (PK) data

The apparent clearance, half-life (*t*_1/2_) and maximal plasma concentration of a representative tricyclic pyrazole, compound **1h**, were determined in vivo in CD1 nu/nu female mice following an intraperitoneal administration of **1h***.* This compound exhibits a good PK profile, with a low plasma clearance (CL = 11 mL/min/kg), long half-life (*T*_1/2_ = 3.9 h) and reaches plasma concentrations above its SRB cellular IC_50_ (*C*_max_ = 5.8 μM; IC_50_ (SRB) = 1.1 μM).

## 3 Conclusion

A triarylimidazole BRAF inhibitor bearing a phenylpyrazole group (**1a**) was identified as an active BRAF inhibitor. Starting from this lead, optimization and SAR investigations led to the identification of the more potent tricyclic 1,4-dihydroindeno[1,2-*c*]pyrazole BRAF inhibitor **1h**, with a favourable PK profile. Replacement of the imidazole core scaffold with furanamide led to compounds with weaker activity against isolated mutant BRAF, but with comparable cellular activity. The triarylimidazole **1j** with a phenylpiperazine ring D is the most promising compound, with nanomolar activity in the mutant BRAF inhibition assay, the cellular pERK inhibition and the mutant BRAF melanoma WM266.4 growth inhibition.

## 4 Experimental

### 4.1 Chemistry

#### 4.1.1 Materials and methods

All starting materials, reagents and solvents for reactions were reagent grade and used as purchased. Chromatography solvents were HPLC grade and were used without further purification. Reactions were monitored by thin layer chromatography (TLC) analysis using Merck Silica Gel 60 F-254 thin layer plates. Flash column chromatography was carried out on Merck Silica Gel 60 (0.015–0.040 mm) or in disposable Isolute Flash Si and Si II silica gel columns. LC-MS analyses were performed on a Micromass LCT/Water’s Alliance 2795 HPLC system using 5 μm Atlantis C18, 50 mm × 2.1 mm columns at 22 °C with the following solvent system: aqueous: water + 0.1% formic acid; organic: 0.1% formic + acetonitrile, at a flow rate of 1 mL/min. Method A: gradient starting with 100% aqueous to 100% organic in 2.5 min at room temperature and a flow rate of 0.6 mL/min or method B gradient starting with 100% aqueous to 100% organic in 5 min at 40 °C (column temperature) at a flow rate of 0.6 mL/min. UV detection was at 215 nm and ionisation was positive or negative ion electrospray. The molecular weight scan range was 50–1000. Samples were injected at 3 μL on a partial loop fill. All automated HPLC purification were performed on Gilson Prep LC modules running on software version 1.71 or 3.0 and on HyperprepHSC18 100 mm × 21.2 mm columns, 12 μm, at a flow rate of 30 mL/min at room temperature using as aqueous phase: water + 0.1% (trifluoroacetic acid) TFA and as organic phase: acetonitrile + 0.1% TFA (UV detector, at 215 and 254 nm). The purity of the final compounds was determined by HPLC as described above and is 95% or higher unless specified otherwise. ^1^H NMR spectra were recorded on either a 250 MHz or a 400 MHz Bruker NMR machine. Accurate Mass Measurement was performed with a Waters Micromass LCT Premier Orthogonal Acceleration Time-of-Flight Mass Spectrometer 4 GHz TDC with LockSpray™ enable mass measurements of 5 ppm or better for *m/z* of 400 or greater and 2 mDa or better for *m/z* of 400 or less. Calibration reference: Wpos_150208.cal or Wneg_150208a.cal. MassLynx v4.1 SCN 633 was the operating software using the in-built elemental composition to report data. Minimum 10 scans are combined across a MS peak.

#### 4.1.2 Synthesis and characterization of intermediates **3**–**9** (Scheme 1)

##### 4.1.2.1 2,4,5-Tribromo-1-methoxymethyl-1*H*-imidazole (**3**)

Tribromo imidazole **2** (6.1 g, 20.0 mmol) was dissolved in dry THF (25 mL) and NaH (60% in mineral oil, 960 mg, 24 mmol) was added portionwise. The solution was cooled to 0 °C under nitrogen atmosphere and MOMBr (3.33 g, 24.0 mmol) was added dropwise over approximately 10 min. The reaction was stirred at room temperature for 2 h or until complete by LC/MS. The solution was diluted with water (100 mL), extracted with EtOAc (3 × 25 mL) and dried (MgSO_4_). The solvent was removed under vacuum to yield 6.8 g of **3** as a white solid. (Yield: 97%). ^1^H NMR (250 MHz, CDCl_3_) *δ* ppm: 5.34 (2H, s), 3.40 (3H, s). LCMS: *t*_R_ = 1.87 min, 346 (M+H)^+^ calcd for C_5_H_5_Br_3_N_2_O.

##### 4.1.2.2 4-(4,5-Dibromo-1-methoxymethyl-1*H*-imidazol-2-yl)-phenol (**4**)

2,4,5-Tribromo-1-methoxymethyl*-1H-*imidazole **3** (2.0 g, 5.72 mmol), 4-hydroxyphenyl-boronic acid (0.821 g, 5.95 mmol), 2 M K_2_CO_3_ solution (6.4 mL) were suspended in a 5:1 mixture of toluene/MeOH (120 mL). The suspension was stirred vigorously whilst de-gassing with nitrogen for 20 min. Pd(PPh_3_)_4_ (0.658 g, 0.57 mmol) was added and the mixture was refluxed overnight. The reaction was cooled to room temperature, diluted with H_2_O (20 mL) and EtOAc (10 mL). The phases were separated. The organic layer was then extracted with 1 M NaOH (3 × 15 mL). Both aqueous layers were separately acidified with 1 M HCl until a white precipitate appeared (pH 6–7). The precipitates were filtered, washed with water, and dried under vacuum to give a total of 0.800 g of **4** as a white solid. (Yield: 39%). ^1^H NMR (250 MHz, DMSO-*d*_6_) *δ* ppm: 7.37 (2H, d, *J* = 8.60 Hz), 6.70 (2H, d, *J* = 8.6 Hz), 5.21 (2H, s), 3.28 (3H, s). LCMS: *t*_R_ = 1.82 min, 360, (M+H)^+^ calcd for C_11_H_10_Br_2_N_2_O_2_. HRMS: (M+H)^+^ calcd for C_11_H_10_Br_2_N_2_O_2_: 360.9187, found: 360.9179.

##### 4.1.2.3 {2-[4-(4,5-Dibromo-1-methoxymethyl-1*H*-imidazol-2-yl)-phenoxy]-ethyl}-dimethyl-amine (**5**)

4-(4,5-Dibromo-1-methoxymethyl*-1H-*imidazol-2-yl)-phenol **4** (300 mg, 0.83 mmol), Cs_2_CO_3_ (649 mg, 1.99 mmol) and 2-dimethylaminoethyl chloride hydrochloride (143 mg, 0.99 mmol) were dissolved in DMF (4 mL). The solution was stirred at 40 °C overnight, then diluted with water (20 mL) and extracted with EtOAc (3 × 10 mL). The organic layer was washed with brine (10 mL), dried (MgSO_4_) and the solvent removed under vacuum to yield 259 mg of **5** as a pale yellow oil. (Yield: 72%). ^1^H NMR (250 MHz, CDCl_3_) *δ* ppm: 7.69 (2H, d, *J* = 8.9 Hz), 7.00 (2H, d, *J* = 9.0 Hz), 5.25 (2H, s), 4.12 (2H, t, *J* = 5.7 Hz), 3.46 (3H, s), 2.76 (2H, t, *J* = 5.7 Hz), 2.36 (6H, s). LCMS: *t*_R_ = 1.37 min, 432 (M+H)^+^ calcd for C_15_H_19_Br_2_N_3_O_2_.

##### 4.1.2.4 {2-[4-(4-Bromo-1-methoxymethyl-5-pyridin-4-yl-1*H*-imidazol-2-yl)-phenoxy]-ethyl}-dimethyl-amine (**6**)

{2-[4-(4,5-Dibromo-1-methoxymethyl*-1H-*imidazol-2-yl)-phenoxy]-ethyl}-dimethyl-amine **5** (208 mg, 0.480 mmol), 4-pyridyl-boronic ester (59 mg, 0.480 mmol), PPh_3_ (12.6 mg, 0.048 mmol), and K_2_CO_3_ (530 mg, 3.840 mmol) were suspended in a 2:1 mixture of DME/H_2_O (9 mL). The suspension was stirred vigorously whilst de-gassing with N_2_ for 20 min before adding Pd(OAc)_2_ (5.4 mg, 0.024 mmol). The mixture was then refluxed for 2 h, cooled to room temperature, acidified to pH 1 with 1 M HCl and washed with EtOAc (3 × 5 mL). The aqueous layer was basified with 2 M NaOH to pH 14 and extracted with EtOAc (3 × 5 mL). This organic layer was dried (MgSO_4_) and the solvent removed under vacuum. The residue was purified by chromatography using a stepped gradient of 5–10% NEt_3_ in EtOAc, then 1:10:89 MeOH/NEt_3_/EtOAc to give 43 mg of **6** as a yellow oil. (Yield: 21%). ^1^H NMR (360 MHz, CDCl_3_) *δ* ppm: 8.70–8.80 (2H, m), 7.75 (2H, d, *J* = 8.9 Hz), 7.56–7.64 (2H, m), 6.97–7.09 (2H, m), 4.96 (2H, s), 4.14 (2H, t, *J* = 5.7 Hz), 3.34 (3H, s), 2.78 (2H, t, *J* = 5.7 Hz), 2.37 (6H, s). LCMS: *t*_R_ = 1.06 min, 431 (M+H)^+^ calcd for C_20_H_23_BrN_4_O_2_. HRMS: (M+H)^+^ calcd for C_20_H_23_BrN_4_O_2_: 431.1082, found: 431.1081.

##### 4.1.2.5 {2-[4-(4-Bromo-5-pyridin-4-yl-1*H*-imidazol-2-yl)-phenoxy]-ethyl}-dimethyl-amine (**7**)

{2-[4-(4-Bromo-1-methoxymethyl-5-pyridin-4-yl-*1H*-imidazol-2-yl)-phenoxy]-ethyl}-dimethyl-amine **6** (70 mg, 0.16 mmol) was dissolved in 1 mL 5 M HCl and the reaction heated at 60 °C for 1 h. The solvents were removed under vacuum to yield 70 mg of imidazole **7** as HCl salt. (Yield: quantitative). ^1^H NMR (360 MHz, MeOH) *δ* ppm 8.82 (2H, d, *J* = 6.8 Hz,), 8.65 (2H, d, *J* = 6.8 Hz,), 8.06 (2H, d, *J* = 9.1 Hz), 7.23 (2H, d, *J* = 9.1 Hz,), 4.39–4.55 (2H, m), 3.57–3.75 (2H, m), 3.02 (7H, s). LCMS: *t*_R_ = 0.94 min, 387 (M+H)^+^ calcd for C_18_H_19_BrN_4_O.

#### 4.1.3 General method for the synthesis of {2-[4-(4-aryl-5-pyridin-4-yl*-1H-*imidazol-2-yl)-phenoxy]-ethyl}-dimethyl-amines

Imidazole **6** or **7** (1 equiv), boronic ester (1.2–2 equiv), PPh_3_ (0.1 equiv), and K_2_CO_3_ (8–10 equiv) were suspended in a 2:1 mixture of DME/H_2_O (3–9 mL). The suspension was stirred vigorously whilst de-gassing with N_2_ for 20 min before adding Pd(OAc)_2_ (0.01 equiv). The mixture was then refluxed for 2 h to overnight, cooled to room temperature, acidified to pH1 with 1 M HCl and washed with EtOAc (3 × 5 mL). The aqueous layer was basified with 2 M NaOH to pH14 and extracted with EtOAc (3 × 5 mL). This organic layer was dried (MgSO_4_) and the solvent removed under vacuum. The residue was purified by preparative HPLC to yield the desired product as TFA salt.

##### 4.1.3.1 {2-[4-(5-{4-[1-(4-Methoxy-benzyl)-1*H*-pyrazol-3-yl]-phenyl}-1-methoxymethyl-4-pyridin-4-yl-1*H*-imidazol-2-yl)-phenoxy]-ethyl}-dimethyl-amine (**8a**)

Using the general coupling procedure with 1-(4-methoxy-benzyl)-3-[4-(4,4,5,5-tetramethyl-[1,3,2]dioxaborolan-2-yl)-phenyl]*-1H-*pyrazole **29a** (77 mg, 0.20 mmol) and MOM protected imidazole **6** (71 mg, 0.16 mmol) gave the title compound which was purified by chromatography on silica gel. Yield = 47 mg (48%). ^1^H NMR (360 MHz, MeOH) *δ* ppm: 8.63 (2H, d, *J* = 5.9 Hz), 7.79 (2H, d, *J* = 8.6 Hz), 7.73 (2H, d, *J* = 8.2 Hz), 7.61 (1H, d, *J* = 2.3 Hz), 7.54 (2H, d, *J* = 5.9 Hz), 7.46 (2H, d, *J* = 8.6 Hz), 7.24 (2H, d, *J* = 8.6 Hz), 7.17 (2H, d, *J* = 8.6 Hz), 6.90 (2H, d, *J* = 8.6 Hz), 6.64 (1H, d, *J* = 2.3 Hz), 5.29 (2H, s), 5.03 (2H, s), 4.22 (2H, t, *J* = 5.4 Hz), 3.78 (3H, s), 3.26 (3H, s), 2.86 (2H, t, *J* = 5.2 Hz), 2.41 (6H, s). LCMS: *t*_R_ = 1.40 min, 615 (M+H)^+^ calcd for C_37_H_38_N_6_O_3_.

##### 4.1.3.2 {2-[4-(5-{4-[1-(4-Methoxy-benzyl)-4-methyl-1*H*-pyrazol-3-yl]-phenyl}-1-methoxy methyl-4-pyridin-4-yl-1*H*-imidazol-2-yl)-phenoxy]-ethyl}-dimethyl-amine (**8b**)

Standard coupling procedure using 1-(4-methoxy-benzyl)-4-methyl-3-[4-(4,4,5,5-tetramethyl-[1,3,2]dioxaborolan-2-yl)-phenyl]*-1H-*pyrazole **29b** (63 mg, 0.16 mol) and MOM protected imidazole **6** (57 mg, 0.13 mmol) gave the title compound which was purified by chromatography on silica gel. Yield = 60 mg (73%). ^1^H NMR (250 MHz, CDCl_3_) *δ* ppm; 8.66 (2H, d, *J* = 5.9 Hz), 7.79 (2H, d, *J* = 8.7 Hz), 7.61 (2H, d, *J* = 11.5 Hz), 7.55 (2H, d, *J* = 11.2 Hz), 7.47 (2H, d, *J* = 6.1 Hz), 7.21 (2H, d, *J* = 8.5 Hz), 7.11 (1H, s), 7.05 (2H, d, *J* = 8.7 Hz), 6.87 (2H, d, *J* = 8.7 Hz), 5.20 (2H, s), 4.95 (2H, s), 4.14 (2H, t, *J* = 5.7 Hz), 3.78 (3H, s), 3.28 (3H, s), 2.78 (2H, t, *J* = 5.6 Hz), 2.36 (6H, s), 2.17 (3H, s). LCMS: *t*_R_ = 1.39 min, 629 (M+H)^+^ calcd for C_38_H_40_N_6_O_3_.

##### 4.1.3.3 3-(4-{2-[4-(2-Dimethylamino-ethoxy)-phenyl]-3-methoxymethyl-5-pyridin-4-yl-3*H*-imidazol-4-yl}-phenyl)-1-(4-methoxy-benzyl)-1*H*-pyrazole-4-carbonitrile (**8c**)

Standard coupling procedure using 1-(4-methoxy-benzyl)-3-[4-(4,4,5,5-tetramethyl-[1,3,2]dioxaborolan-2-yl)-phenyl]*-1H-*pyrazole-4-carbonitrile **37** (65 mg, 0.16 mol) and MOM protected imidazole **6** (57 mg, 0.13 mmol) gave the title compound which was purified by chromatography on silica gel. Yield = 61 mg (73%). ^1^H NMR (250 MHz, CDCl_3_) *δ* ppm: 8.62 (2H, d, *J* = 6.1 Hz), 7.82 (2H, d, *J* = 8.5 Hz), 7.72 (2H, d, *J* = 8.8 Hz), 7.63 (1H, s), 7.54 (2H, d, *J* = 8.5 Hz), 7.39 (2H, d, *J* = 6.1 Hz), 7.17 (2H, d, *J* = 8.8 Hz), 6.98 (2H, d, *J* = 8.8 Hz), 6.83 (2H, d, *J* = 8.8 Hz), 5.18 (2H, s), 4.94 (2H, s), 4.07 (3H, t, *J* = 5.7 Hz), 3.73 (3H, s), 3.21 (3H, s), 2.70 (2H, t, *J* = 5.6 Hz), 2.29 (6H, s). LCMS: *t*_R_ = 1.50 min, 640 (M+H)^+^ calcd for C_38_H_37_N_7_O_3_.

##### 4.1.3.4 [2-(4-{4-[1-(4-Methoxy-benzyl)-4,5-dihydro-1*H*-benzo[*g*]indazol-7-yl]-1-methoxy methyl-5-pyridin-4-yl-1*H*-imidazol-2-yl}-phenoxy)-ethyl]-dimethyl-amine (**8e**)

The standard coupling procedure using 1-(4-methoxybenzyl)-7-(4,4,5,5-tetramethyl-1,3,2-dioxaborolan-2-yl)-4,5-dihydro*-1H-*benzo[*g*]indazole **40** (37 mg, 0.09 mmol) and imidazole **6** (31 mg, 0.07 mmol) resulted in **8e** (13 mg, 29%). ^1^H NMR (250 MHz, CDCl_3_) *δ* ppm: 8.68 (2H, br s), 7.67–7.86 (3H, m), 7.36–7.58 (3H, m), 7.16–7.34 (5H, m), 7.06 (2H, d, *J* = 10.1 Hz), 6.88 (1H, d, *J* = 8.7 Hz), 5.25 (2H, s), 4.96 (2H, s), 4.15 (2H, t, *J* = 5.7 Hz), 3.78 (3H, s), 3.29 (3H, s), 2.65–2.90 (6H, m), 2.37 (6H, s). LCMS: *t*_R_ = 1.41 min, 641 (M+H)^+^ calcd for C_39_H_40_N_6_O_3_.

##### 4.1.3.5 [2-(4-{4-[1-(4-Methoxy-benzyl)-1*H*-benzo[*g*]indazol-7-yl]-1-methoxymethyl-5-pyridin-4-yl-1*H*-imidazol-2-yl}-phenoxy)-ethyl]-dimethyl-amine (**8f**)

The standard coupling procedure using 1-(4-methoxy-benzyl)-7-(4,4,5,5-tetramethyl-[1,3,2]dioxaborolan-2-yl)*-1H-*benzo[*g*]indazole **43** (53 mg, 0.13 mmol) and imidazole **6** (71 mg, 0.16 mmol) resulted in **8f** (33 mg, crude). Taken into next step without further purification. LCMS: *t*_R_ = 1.47 min, 639 (M+H)^+^ calcd for C_39_H_38_N_6_O_3_.

##### 4.1.3.6 7-{2-[4-(2-Dimethylamino-ethoxy)-phenyl]-1-methoxymethyl-5-pyridin-4-yl-1*H*-imidazol-4-yl}-2,4,4a,5-tetrahydro-indeno[1,2-*c*]pyridazin-3-one (**8g**)

The standard coupling procedure using 7-(4,4,5,5-tetramethyl-[1,3,2]dioxaborolan-2-yl)-2,4,4a,5-tetrahydro-indeno[1,2-*c*]pyridazin-3-one **26** (60 mg, 0.19 mmol) and imidazole **6** (99 mg, 0.23 mmol) resulted in **8g** (65 mg, 63%). ^1^H NMR (360 MHz, CDCl_3_) *δ* ppm: 8.72 (2H, d, *J* = 6.1 Hz), 8.48 (1H, s), 7.79 (2H, d, *J* = 8.9 Hz), 7.67 (1H, br s), 7.56 (1H, d, *J* = 7.9 Hz), 7.47 (2H, d, *J* = 6.1 Hz), 7.40 (1H, d, *J* = 8.4 Hz), 7.06 (2H, d, *J* = 8.9 Hz), 4.95 (2H, s), 4.16 (2H, t, *J* = 5.7 Hz), 3.10–3.43 (5H, m), 2.91 (1H, dd, *J* = 16.6, 7.3 Hz), 2.81 (2H, t, *J* = 5.7 Hz), 2.70 (1H, dd, *J* = 16.3, 5.9 Hz), 2.29–2.42 (7H, m). LCMS: *t*_R_ = 1.11 min, 537 (M+H)^+^ calcd for C_31_H_32_N_6_O_3_.

##### 4.1.3.7 {2-[4-(5-{4-[4-Chloro-1-(4-methoxy-benzyl)-1*H*-pyrazol-3-yl]-phenyl}-4-pyridin-4-yl-1*H*-imidazol-2-yl)-phenoxy]-ethyl}-dimethyl-amine (**9d**)

Standard coupling procedure using 4-chloro-1-(4-methoxy-benzyl)-3-[4-(4,4,5,5-tetramethyl-[1,3,2]dioxaborolan-2-yl)-phenyl]*-1H-*pyrazole **33** (30 mg, 0.071 mmol) and deprotected imidazole hydrochloride **7** (27 mg, 0.059 mmol) gave the title compound **9d** which was purified by chromatography on silica gel. Yield = 18 mg (51%). ^1^H NMR (360 MHz, MeOH) *δ* ppm: 8.41 (2H, d, *J* = 5.9 Hz), 7.90–7.96 (4H, m), 7.77 (1H, s), 7.51–7.58 (4H, m), 7.26 (2H, d, *J* = 9.1 Hz), 7.06 (2H, d, *J* = 9.1 Hz), 6.90 (2H, d, *J* = 8.6 Hz), 5.24 (2H, s), 4.16 (2H, t, *J* = 5.4 Hz), 3.76 (3H, s), 2.83 (2H, t, *J* = 5.4 Hz), 2.39 (6H, s). LCMS: *t*_R_ = 1.41 min, 605 (M+H)^+^ calcd for C_35_H_32_ClN_5_O_2_.

#### 4.1.4 Synthesis and characterization of intermediates **11**–**16** (Scheme 2)

##### 4.1.4.1 1-Methoxyimino-indan-5-carbaldehyde (**11**)

5-Bromo-2,3-dihydro-1*H*-inden-1-one *O*-methyl oxime **10** (3.71 g, 15.45 mmol) was dissolved in dry THF (75 mL). Solution was de-gassed over N_2_ and then cooled to −78 °C. *n*BuLi (1.6 M in THF, 10.63 mL, 17.0 mmol) was added dropwise. Stirring at −78 °C was continued for a further 10 min, before adding dry DMF (1.32 mL). Reaction was allowed to gradually warm to room temperature over 3 h. Reaction was then quenched with saturated NaHCO_3_ and THF was evaporated under vacuum. Residue was dissolved in EtOAc and washed with water, brine and dried (MgSO_4_), and solvent removed to leave a brown oil. Purification was achieved by Flash Column Chromatography on Silica gel, eluting in 5–10% EtOAc/heptane. Yield: 2.27 g (78%). ^1^H NMR (250 MHz, CDCl_3_) *δ* ppm: 9.95 (1H, s), 7.64–7.80 (3H, m), 3.95 (3H, s), 2.98–3.09 (2H, m), 2.81–2.93 (2H, m). LCMS: *t*_R_ = 1.84 min, 190 (M+H)^+^ calcd for C_11_H_11_NO_2_.

##### 4.1.4.2 5-(1-Hydroxy-2-pyridin-4-yl-2-TBDMS-hydroxy-propyl)-indan-1-one *O*-methyl-oxime (**12**)

4-(*tert*-Butyl-dimethyl-silanyloxymethyl)-pyridine (2.80 g, 12.5 mmol) was dissolved in dry THF (20 mL) and the solution was de-gassed over N_2_. Reaction was cooled to −40 °C (MeCN:Cardice) and the LDA (2 M sol, 7.43 mL, 11.4 mmol) was added dropwise. Reaction was stirred at −40 °C under N_2_ for 30 min, whereupon a de-gassed solution of 1-methoxyimino-indan-5-carbaldehyde **11** (2.16 g, 11.4 mmol) in THF (20 mL), was added dropwise and the reaction mixture gradually allowed to warm to room temperature over 3 h. Reaction mixture was quenched with satd Na_2_HCO_3_ and the THF was removed under vacuum. Residue was taken up in EtOAc, washed with satd Na_2_HCO_3_, water, brine and dried (MgSO_4_). Solvent was removed under vacuum to leave a yellow oil which was purified by Flash Column Chromatography on silica gel, eluting the desired product in 50–100% EtOAc/heptane. Yield: 4.52 g (96%) as a yellow oil. ^1^H NMR (250 MHz, CDCl_3_) *δ* ppm: 8.60–8.72 (2H, m), 7.71–7.82 (1H, m), 7.10–7.36 (4H, m), 4.69–5.00 (2H, m), 4.13–4.20 (3H, m), 3.00–3.21 (4H, m), 0.96–1.10 (9H, m), −0.13 (6H, m). LCMS: *t*_R_ = 1.85 min 413 (M+H)^+^ calcd for C_23_H_30_N_2_O_3_Si.

##### 4.1.4.3 5-(1,2-Dihydroxy-2-pyridin-4-yl-ethyl)-indan-1-one *O*-methyl-oxime (**13**)

5-(1-Hydroxy-2-pyridin-4-yl-2-TBDMS-hydroxy-propyl)-indan-1-one *O*-methyl-oxime **12** (1.24 g, 3.01 mmol) was dissolved in dry THF (50 mL) and TBAF (1 M in THF) (4.81 mL, 4.81 mmol) was added. Reaction mixture was stirred at room temperature over night. Solvent was removed under vacuum and residue was purified by Flash Column Chromatography on silica gel, eluting the desired product in 5% MeOH/DCM as a colourless solid. Yield: 797 mg (89%). ^1^H NMR (250 MHz, CDCl_3_) *δ* ppm: 8.39 (1H, dt, *J* = 4.30, 1.81 Hz), 7.54 (1H, d, *J* = 8.07 Hz), 6.94–7.13 (4H, m), 4.93–5.17 (1H, m), 4.62–4.78 (1H, m), 3.98 (3H, s), 2.77–3.02 (4H, m). LCMS: *t*_R_ = 1.06 min, 299 (M+H)^+^ calcd for C_17_H_18_N_2_O_3_.

##### 4.1.4.4 1-(1-Methoxyimino-indan-5-yl)-2-pyridin-4-yl-ethane-1,2-dione (**14**)

DMSO (705 μl, 9.95 mmol) was dissolved in dry DCM (6.4 mL) and cooled to −78 °C under N_2_. Oxalyl chloride (653 μL, 7.50 mmol) was then added dropwise and the solution was stirred for 20 min. A solution of diol **13** (671 mg, 2.25 mmol) in dry DCM (6.4 mL) and DMSO (959 μL, 6.0 mmol) was added dropwise at −78 °C and the solution was stirred for 30 min. Triethylamine (1.94 mL, 14.0 mmol) was then added dropwise and the reaction mixture was gradually allowed to warm to room temperature over 3 h. Reaction mixture was diluted with water and extracted with DCM (3 × 20 mL), washed with brine, dried (MgSO_4_) and evaporated to dryness under vacuum to leave a yellow solid. Purified by Flash column chromatography on silica gel, eluting the desired product in 50–100% EtOAc/heptane. Yield: 1.15 g (quantitative) as a yellow solid. ^1^H NMR (250 MHz, CDCl_3_) *δ* ppm: 8.78–9.06 (2H, m), 7.71–7.97 (5H, m), 4.04 (3H, s), 3.04–3.18 (2H, m), 2.81–3.02 (2H, m). LCMS: *t*_R_ = 2.07 min, 295 (M+H)^+^ calcd for C_17_H_14_N_2_O_3_.

##### 4.1.4.5 5-{2-[2-(4-(4-methylpiperazin-1-yl)-5-(pyridine-4-yl)-1*H*-imidazol-4-yl)]}-1*H*-inden-1-one-*O*-methyl-oxime (**15a**)

4-(4′-*N*-Methyl-pyrazinyl)-benzaldehyde (135 mg, 0.66 mmol) was dissolved in acetic acid (3 mL), diketone **14** (150 mg, 0.51 mmol) and ammonium acetate (393 mg, 5.10 mmol) was added. The reaction mixture was heated to 100 °C and stirred for 1 h. Reaction mixture was basified with NH_3_, and diluted with water. Aqueous was extracted with DCM (3 × 10 mL), dried (MgSO_4_) filtered and evaporated under vacuum. Purification was carried out by Flash column chromatography on silica gel, eluting the desired product in 5–10% MeOH/DCM, to yield imidazole **15a.** Yield: 93.5 mg (38%). ^1^H NMR (250 MHz, MeOD) *δ* ppm: 8.43 (2H, dd, *J* = 4.7, 1.5 Hz), 7.89 (2H, d, *J* = 9.0 Hz), 7.69 (1H, d, *J* = 8.1 Hz), 7.48–7.58 (3H, m), 7.38 (1H, s), 7.08 (2H, d, *J* = 9.0 Hz), 3.97 (3H, s), 3.25–3.35 (4H, m), 3.02–3.08 (2H, m), 2.88–2.96 (2H, m), 2.68–2.78 (4H, m), 2.44 (3H, s). LCMS: *t*_R_ = 1.21 min, 479 (M+H)^+^ calcd for C_29_H_30_N_6_O.

##### 4.1.4.6 5-{2-[4-(2-Dimethylamino-ethoxy)-phenyl]-5-pyridin-4-yl-1*H*-imidazol-4-yl}-indan-1-one *O*-methyl-oxime (**15b**)

4-(2-Dimethylamino-ethoxy)-benzaldehyde (367 mg, 1.90 mmol) was reacted with diketone **14** (428 mg, 1.45 mmol) as described for compound **15a** to give *O*-methyl oxime **15b** (197 mg, 29%) as a yellow oil. ^1^H NMR (250 MHz, MeOD) *δ* ppm: 8.42 (2H, dd, *J* = 4.8, 1.29 Hz), 7.90–7.97 (2H, m), 7.68 (1H, d, *J* = 8.1 Hz), 7.45–7.57 (3H, m), 7.37 (1H, dd, *J* = 8.1, 1.2 Hz), 7.07 (2H, d, *J* = 9.0 Hz), 4.16 (2H, t, *J* = 5.4 Hz), 3.96 (3H, s), 2.99–3.10 (2H, m), 2.85–2.95 (2H, m), 2.80 (2H, t, *J* = 5.5 Hz), 2.36 (s, 6H). LCMS: *t*_R_ = 1.19 min, 468 (M+H)^+^ calcd for C_28_H_29_N_5_O_2_.

##### 4.1.4.7 (*E*)-*tert*-Butyl 4-(4-(1-(methoxyimino)-2,3-dihydro-1*H*-inden-5-yl)-5-(pyridin-4-yl)-1*H*-imidazol-2-yl)piperidine-1-carboxylate (**15c**)

4-Formyl-piperidine-1-carboxylic acid *tert*-butyl ester (141 mg 0.66 mmol) was reacted with diketone **14** (150 mg, 0.51 mmol) as described for **15a** to give *O*-methyl oxime **15c** (229 mg, 92%). ^1^H NMR (250 MHz, MeOD) *δ* ppm: 8.41 (2H, d, *J* = 5.94 Hz), 7.67 (1H, d, *J* = 8.22 Hz), 7.45 (3H, d, *J* = 12.03 Hz), 7.32 (1H, d, *J* = 8.22 Hz), 4.22 (2H, d, *J* = 12.94 Hz), 3.96 (3H, s), 2.83–3.11 (7H, m), 1.94–2.07 (2H, m), 1.71–1.92 (2H, m), 1.48 (s, 9H). LCMS: *t*_R_ = 1.55 min, 488 (M+H)^+^ calcd for C_28_H_33_N_5_O_3_.

##### 4.1.4.8 5-(2-(4-(4-Methylpiperazin-1-yl)phenyl)-5-(pyridin-4-yl)-1*H*-imidazol-4-yl)-2,3-dihydro-1*H*-inden-1-one (**16a**)

5-{2-[2-(4-(4-Methylpiperazin-1-yl)-5-(pyridine-4-yl)-*1H*-imidazol-4-yl)]}-*1H*-inden-1-one-*O*-methyl-oxime **15a** (93.5 mg, 0.20 mmol) was dissolved in dioxane (4 mL) and 5 M HCl (2 mL) was added. The reaction was heated to 80 °C overnight. Solvents were evaporated under vacuum and residue was purified by Flash column chromatography on silica gel, eluting the desired product in 5–50% MeOH/DCM to give ketone **16a**. Yield: 87 mg (99%). ^1^H NMR (250 MHz, MeOD) *δ* ppm: 8.83 (2H, br s), 8.05–8.27 (4H, m), 7.49–7.96 (3H, m), 7.30 (2H, d, *J* = 7.3 Hz), 4.17 (2H, br s), 3.55–3.78 (5H, m), 3.34–3.46 (3H, m), 2.91–3.06 (4H, m), 2.68–2.84 (1H, m). LCMS: *t*_R_ = 1.08 min, 450 (M+H)^+^ calcd for C_28_H_28_N_6_O.

##### 4.1.4.9 {2-[4-(5-Indanyl-1-one)-5-pyridin-4-yl-1*H*-imidazol-2-yl]-phenoxy]-ethyl}-dimethyl-amine (**16b**)

5-{2-[4-(2-Dimethylamino-ethoxy)-phenyl]-5-pyridin-4-yl-*1H*-imidazol-4-yl}-indan-1-one *O*-methyl-oxime **15b** (164 mg, 0.35 mmol) was treated as described for compound **16a** to give ketone **16b** (36 mg, 23%) as a yellow oil. ^1^H NMR (250 MHz, MeOD) *δ* ppm: 8.47 (2H, d, *J* = 6.1 Hz), 7.97 (2H, d, *J* = 8.8 Hz), 7.72 (2H, s), 7.55 (3H, br s), 7.10 (2H, d, *J* = 8.8 Hz), 4.19 (2H, t, *J* = 5.4 Hz), 3.15–3.40 (2H, m), 2.62–2.91 (4H, m), 2.38 (6H, s). LCMS: *t*_R_ = 1.08 min, 439 (M+H)^+^ calcd for C_27_H_26_N_4_O_2_.

##### 4.1.4.10 *tert*-Butyl 4-(4-(1-oxo-2,3-dihydro-1*H*-inden-5-yl)-5-(pyridin-4-yl)-1*H*-imidazol-2-yl)piperidine-1-carboxylate (**16c**)

*tert*-Butyl 4-(4-(1-(methoxyimino)-2,3-dihydro*-1H-*inden-5-yl)-5-(pyridin-4-yl)*-1H-*imidazol-2-yl)piperidine-1-carboxylate **15c** (229 mg, 0.47 mmol) was dissolved in 5 M HCl (2 mL) and dioxane (4 mL) and heated at 80 °C overnight. All solvents were removed under vacuum to leave a brown oil (LCMS: *t*_R_ = 0.86 min, 359 (M+H)^+^ calcd for C_22_H_22_N_4_O). This was dissolved in THF (15 mL) and water (2 mL) before adding DIPEA (90 μL, 0.52 mmol) and (BOC)_2_O (256 mg, 1.17 mmol). After stirring at room temperature overnight, the THF was evaporated, further water added and extracted with DCM (×3). The combined organic phases were dried (MgSO_4_), filtered and evaporated to leave an oil which was purified by Flash column chromatography on silica gel, eluting the desired compound with 0–10% MeOH/DCM. Yield = 122 mg (57%). ^1^H NMR (250 MHz, MeOD) *δ* ppm: 8.45 (2H, br s), 7.57–7.79 (2H, m), 7.34–7.54 (3H, m), 4.22 (2H, d, *J* = 12.6 Hz), 3.15–3.40 (2H, m), 2.86–3.08 (3H, m), 2.72 (2H, t), 1.95–2.09 (2H, m), 1.84 (2H, t, *J* = 12.0 Hz), 1.48 (9H, s). LCMS: *t*_R_ = 1.44 min, 459 (M+H)^+^ calcd for C_27_H_30_N_4_O_3_.

#### 4.1.5 Synthesis and characterization of intermediates **17**–**22** (Scheme 4)

##### 4.1.5.1 Methyl, 2,3-dibromofuranyl-carboxylate (**17**)

To a solution of the 2,3-dibromofuran-5-yl carboxylic acid (4 g, 14.8 mmol) in DCM (60 mL) was added oxalyl chloride (3.88 mL, 44.5 mmol) under nitrogen. The reaction mixture was cooled to 0 °C and 0.2 mL of DMF added. The reaction was stirred at room temperature for 4 h, solvent was removed under vacuum and azeotroped with DCM. The crude was dissolved in DCM (40 mL) and MeOH added at 0 °C. The reaction was stirred at room temperature for 1 h and solvent removed under vacuum*.* Yielded product was obtained as a pure solid (3.85 g) in 91% yield. ^1^H NMR (250 MHz, CDCl_3_-*d*) *δ* ppm: 7.11 (1H, s), 3.83 (3H, s).

##### 4.1.5.2 Methyl, 2-(4-pyridyl),3-bromofuranyl-carboxylate (**18**)

Dry DMF (150 mL) was added to a dry mixture of the dibromofuran **17** (5.68 g, 20.0 mmol), 4-pyridyl boronic acid (2.7 g, 22.0 mmol), Cs_2_CO_3_ (19 g, 60 mmol), AsPh_3_ (0.610 g, 2 mmol), and (PPh_3_)_2_PdCl_2_ (1.12 g, 1.6 mmol). This solution was de-gassed with nitrogen for 20 min before being heated to 90 °C overnight. The majority of DMF was then removed under vacuum and the crude diluted with EtOAc. The crude organic was washed with NaHCO_3_ (1% aqueous) and dried (MgSO_4_). Purification followed using flash column chromatography on silica gel eluting with EtOAc/heptane to yield 2.12 g of product (37%) as an off-white solid. ^1^H NMR (250 MHz, CDCl_3_-*d*) *δ* ppm: 8.73 (2H, d, *J* = 6.2 Hz), 7.93–7.99 (2H, m), 7.30 (1H, s), 3.95 (3H, s). LCMS: *t*_R_ = 1.35 min, 282 (M+H)^+^ calcd for C_11_H_8_BrNO_3_.

##### 4.1.5.3 Methyl 4-(1-(methoxyimino)-2,3-dihydro-1*H*-inden-5-yl)-5-(pyridin-4-yl)furan-2-carboxylate (**19**)

Dry DMF (150 mL) was added to a dry mixture of **18** (0.590 g, 2.1 mmol), 1-(methoxyimino)-2,3-dihydro-*1H*-inden-5-ylboronic acid (0.512 g, 2.5 mmol), Cs_2_CO_3_ (2.05 g, 6.3 mmol), AsPh_3_ (0.640 g, 2.1 mmol), and (PPh_3_)_2_PdCl_2_ (1.05 g, 1.5 mmol). This solution was de-gassed with nitrogen for 20 min before being heated to 90 °C overnight. The majority of DMF was then removed under vacuum and the crude diluted with EtOAc. The crude organic was washed with 1% aqueous NaHCO_3_ and dried (MgSO_4_). Purification followed using flash column chromatography on silica gel eluting with EtOAc/heptane to yield 0.519 g of product (69%) as an off-white solid. ^1^H NMR (250 MHz, CDCl_3_-*d*) *δ* ppm: 8.48 (2H, d, *J* = 4.8 Hz), 7.66 (1H, d, *J* = 8.1 Hz), 7.37–7.42 (2H, m), 7.15–7.23 (3H, m), 3.93 (3H, s), 3.87 (3H, s), 2.94–3.02 (2H, m), 2.81–2.90 (2H, m). LCMS: *t*_R_ = 1.84 min, 363 (M+H)^+^ calcd for C_21_H_18_N_2_O_4_.

##### 4.1.5.4 2-(4-Pyridyl)-3-(5-indanyl-*N*-*O*-methyl-oxime)-furanyl-5-carboxylic acid (**20**)

To a solution of **19** (335 mg, 0.93 mmol) in 5 mL THF/MeOH (4:1) was added 2 M aqueous NaOH (0.95 mL, 1.90 mmol). The reaction was stirred vigorously at room temperature for 90 min and solvent was removed under vacuum to yield 220 mg product (68%) as a white solid. ^1^H NMR (250 MHz, DMSO-*d*_6_) *δ* ppm: 8.26 (2H, d, *J* = 6.8 Hz), 7.97 (2H, d, *J* = 6.5 Hz), 7.65 (1H, d, *J* = 8.1 Hz), 7.53 (2H, s), 7.38 (1H, d, *J* = 7.8 Hz), 3.88 (3H, s), 2.96–3.08 (2H, m), 2.76–2.88 (2H, m). LCMS: *t*_R_ = 1.40 min, 349 (M+H)^+^ calcd for C_20_H_16_N_2_O_4_.

##### 4.1.5.5 4-(1-Oxo-2,3-dihydro-1*H*-inden-5-yl)-5-(pyridin-4-yl)furan-2-carboxylic acid (**21**)

To a solution of starting acid **20** (835 mg, 2.41 mmol) dioxane/acetone (8 mL:1.5 mL) was added 5 mL aqueous hydrochloric acid. The reaction was heated at 90 °C for 90 min. Solvent was removed under vacuum to give 1.41 g of pure product (quantitative, some NaCl present). ^1^H NMR (250 MHz, MeOD) *δ* ppm: 9.48 (2H, d, *J* = 7.8 Hz), 8.70 (2H, d, *J* = 7.7 Hz), 8.45–8.54 (2H, m), 8.24–8.33 (2H, m), 3.90–3.95 (2H, m), 3.42–3.50 (2H, m). HRMS: (M+H)^+^ calcd for C_19_H_13_NO_4_: 320.0923, found: 320.0915.

##### 4.1.5.6 2-(4-Pyridyl)-3-(5-indanyl-oxime)-furanyl-5-carbonyl-4-*N*-methyl-piperidine (**22a**)

To a solution of acid **21** (300 mg, 0.95 mmol) in DMF was added DIC (13 mg, 1.05 mmol), methyl piperazine (105 mg, 1.05 mmol), HOBt (141 mg, 1.05 mmol), and TEA (146 μL, 1.05 mmol). The reaction was heated to 60 °C for 4 h. Solvent was removed under vacuum and the residue was purified by chromatography on silica gel eluting with DCM/MeOH. This yielded 150 mg (40%) of pure product as a white powder. LCMS: *t*_R_ = 0.97 min, 402 (M+H)^+^ calcd for C_24_H_23_N_3_O_3_.

##### 4.1.5.7 5-{5-[4-(2-Dimethylamino-ethyl)-piperidine-1-carbonyl]-2-pyridin-4-yl-furan-3-yl}-indan-1-one (**22b**)

Acid **21** (250 mg, 0.78 mmol) was coupled with dimethyl-(2-piperazin-1-yl-ethyl)-amine (135 mg, 0.86 mmol) as described for **22a** to give **22b**. Yield = 176 mg (49%). ^1^H NMR (360 MHz, CDCl_3_) *δ* ppm: 8.58 (2H, d, *J* = 6.4 Hz), 7.81 (1H, d, *J* = 7.9 Hz), 7.52 (1H, s), 7.41 (1H, d, *J* = 7.9 Hz), 7.37 (2H, d, *J* = 6.1 Hz), 7.14 (1H, s), 3.91 (4H, br s), 3.12–3.25 (2H, m), 2.69–2.85 (2H, m), 2.40–2.69 (8H, m), 2.28 (6H, s). LCMS: *t*_R_ = 1.05 min, 459 (M+H)^+^ calcd for C_27_H_30_N_4_O_3_.

##### 4.1.5.8 5-{5-[4-Morpholine-1-carbonyl]-2-pyridin-4-yl-furan-3-yl}-indan-1-one (**22c**)

Acid **21** (766 mg; 2.4 mmol) was coupled with morpholine (0.23 mL; 2.6 mmol) as described for **22a** to give **22c**. Yield = 520 mg (56%). ^1^H NMR (250 MHz, CDCl_3_) *δ* ppm: 8.58 (2H, dd, *J* = 4.6, 1.6 Hz), 7.81 (1H, d, *J* = 7.9 Hz), 7.52 (1H, s), 7.33–7.44 (3H, m), 7.18 (1H, s), 3.75–4.07 (8H, m), 3.13–3.23 (2H, m), 2.72–2.81 (2H, m). LCMS: *t*_R_ = 1.29 min, 389 (M+H)^+^ calcd for C_23_H_20_N_2_O_4_.

##### 4.1.5.9 *tert*-butyl 4-(4-(1-oxo-2,3-dihydro-1*H*-inden-5-yl)-5-(pyridin-4-yl)furan-2-carbonyl)piperazine-1-carboxylate (**22d**)

Acid **21** (385 mg; 1.21 mmol) was coupled with *N*-Boc piperazine (248 mg; 1.33 mmol) as described for **22a** to give **22d** and was used in the next step without further purification. LCMS: *t*_R_ = 1.71 min, 488 (M+H)^+^ calcd for C_28_H_29_N_3_O_5_.

#### 4.1.6 Synthesis and characterization of intermediates **24**–**33** (Scheme 5)

##### 4.1.6.1 (5-Bromo-1-oxo-indan-2-yl)-acetic acid ethyl ester (**24**)

LDA (2 M in hexane, 5.21 mL, 10.42 mmol) was mixed with THF (5 mL) and cooled to −78 °C. 5-Bromo-indan-1-one (2.0 g, 9.48 mmol) in THF (20 mL) was added dropwise under a stream of nitrogen and the solution was stirred for 30 min. Bromo-acetic acid ethyl ester (1.74 g, 10.42 mmol) in HMPA (1.5 mL) and THF (5 mL) was added dropwise to the reaction mixture, which was then allowed to warm up to room temperature overnight. The reaction was quenched with NH_4_Cl saturated solution (50 mL) and extracted with EtOAc (3 × 50 mL), the organic layer was dried (MgSO_4_), filtered and the solvent removed under vacuum. The residue was purified by chromatography using a stepped gradient of 0–8% EtOAc in heptane to yield **24** as a white solid. Yield: 577 mg, 20%. ^1^H NMR (360 MHz, CDCl_3_) *δ* ppm: 7.59–7.68 (2H, m), 7.53 (1H, d, *J* = 9.1 Hz), 4.14 (2H, q, *J* = 7.0 Hz), 3.44 (1H, dd, *J* = 17.1, 7.8 Hz), 2.83–3.06 (3H, m), 2.62–2.73 (1H, m), 1.23 (3H, t, *J* = 7.0 Hz). LCMS: *t*_R_ = 2.07 min, 297 (M+H)^+^ calcd for C_13_H_13_BrO_3_. HRMS: (M+H)^+^ calcd for C_13_H_13_BrO_3_: 297.0126, found: 297.0119.

##### 4.1.6.2 7-Bromo-2,4,4a,5-tetrahydro-indeno[1,2-*c*]pyridazin-3-one (**25**)

Intermediate **24** (250 mg, 0.84 mmol) was dissolved in EtOH (3 mL) and NH_2_NH_2_·H_2_O (0.15 mL, 4.21 mmol) was added in EtOH (1 mL) and stirred at room temperature overnight. A precipitate appeared which was filtered and washed with EtOH (10 mL) to yield compound **25** as a white solid. Yield: 134 mg, 60%. ^1^H NMR (360 MHz, CDCl_3_) *δ* ppm: 8.54 (1H, br s), 7.53–7.60 (2H, m), 7.47–7.51 (1H, m), 3.37–3.47 (1H, m), 3.15–3.27 (1H, m), 2.93 (1H, dd, *J* = 16.6, 7.5 Hz), 2.75 (1H, dd, *J* = 16.6, 6.1 Hz), 2.37 (1H, t, *J* = 16.3 Hz). LCMS: *t*_R_ = 1.65 min, 265 (M+H)^+^ calcd for C_11_H_8_BrN_2_O_3_.

##### 4.1.6.3 7-(4,4,5,5-Tetramethyl-[1,3,2]dioxaborolan-2-yl)-2,4,4a,5-tetrahydro-indeno[1,2-*c*]pyridazin-3-one (**26**)

Bromo intermediate **25** (145 mg, 0.55 mmol), potassium acetate (160 mg, 1.64 mmol), bis(pinacolato)diboron (208 mg, 0.82 mmol) and 1,1-bis(diphenylphosphino)ferrocene-palladium(II)dichloride (20 mg, 0.05 mmol) were dissolved in dry DMF (3 mL). The reaction was heated to 80 °C in a sealed tube for 2 h, quenched with H_2_O (15 mL), extracted into EtOAc (3 × 10 mL) and dried (Na_2_SO_4_). Organic solvent was removed in vacuo. The residue was purified by chromatography using a stepped gradient of 10–50% EtOAc in heptane to yield **26** as a white solid (88 mg, 51%). ^1^H NMR (360 MHz, CDCl_3_) *δ* ppm: 8.47 (1H, s), 7.83 (1H, s), 7.66–7.82 (2H, m), 3.44 (1H, dd, *J* = 16.3, 8.6 Hz), 3.11–3.26 (1H, m), 2.93 (1H, dd, *J* = 16.3, 7.3 Hz), 2.75 (1H, dd, *J* = 16.3, 5.9 Hz), 2.37 (1H, t, *J* = 16.3 Hz), 1.37 (12H, s). LCMS: *t*_R_ = 1.89 min, 313 (M+H)^+^ calcd for C_17_H_22_BN_2_O_3_. HRMS: (M)^+^ calcd for C_17_H_21_BN_2_O_3_: 312.1760, found: 312.1747.

##### 4.1.6.4 3-(4-Bromo-phenyl)-1-(4-methoxy-benzyl)-1*H*-pyrazole (**28a**)

3-(4-Bromophenyl)pyrazole **27a** (223 mg, 1 mmol) was dissolved in anhydrous DMF (2.2 mL) and added to 60% NaH in mineral oil (44 mg, 1.1 mmol) previously washed with diethyl ether. After stirring for 20 min at room temperature, 4-methoxybenzyl chloride (0.15 mL; 1.1 mmol) was added and stirring continued at room temperature for a further 4 h. Water (5 mL) was then added and the emulsion extracted with EtOAc (2 × 3 mL). The combined organic phases were washed with saturated sodium bicarbonate (3 × 2 mL) and dried (MgSO_4_). After filtration and evaporation, the oil crystallised to give the product (352 mg, 100%). ^1^H NMR (250 MHz, CDCl_3_) *δ* ppm: 7.66–7.73 (2H, m), 7.51 (2H, d, *J* = 8.7 Hz), 7.33 (1H, d, *J* = 2.3 Hz), 7.23 (2H, d, *J* = 8.8 Hz), 6.89 (2H, d, *J* = 8.7 Hz), 6.53 (1H, d, *J* = 2.3 Hz), 5.29 (2H, s), 3.81 (3H, s). The product was used in the next step without further purification.

##### 4.1.6.5 3-(4-Bromo-phenyl)-1-(4-methoxy-benzyl)-4-methyl-1*H*-pyrazole (**28b**)

4-Methyl-3-(4-bromophenyl)pyrazole **27b** (474 mg, 2.0 mmol) was dissolved in anhydrous DMF (5 mL) and added to 60% NaH in mineral oil (88 mg, 2.2 mmol) previously washed with heptane. After stirring for 20 min at room temperature, 4-methoxybenzyl chloride (0.3 mL, 2.2 mmol) was added and stirring continued at room temperature for a further 3.5 h. Water (10 mL) was then added and the emulsion extracted with EtOAc (2 × 10 mL). The combined organic phases were washed with saturated sodium bicarbonate (3 × 20 mL) and dried (MgSO_4_). After filtration and evaporation, the oil crystallised to give the product (697 mg, 98%). ^1^H NMR (360 MHz, CDCl_3_) *δ* ppm: 7.58–7.63 (2H, m), 7.56 (2H, d), 7.26 (2H, d, *J* = 8.6 Hz), 7.17 (1H, br s), 6.92 (2H, d, *J* = 8.6 Hz), 5.25 (2H, s), 3.84 (3H, s), 2.21 (3H, s). LCMS: *t*_R_ = 2.48 min, 357 (M+H)^+^ calcd for C_18_H_17_BrN_2_O. HRMS: (M+H)^+^ calcd for C_18_H_17_BrN_2_O: 357.0602, found: 357.0613.

##### 4.1.6.6 1-(4-Methoxy-benzyl)-3-[4-(4,4,5,5-tetramethyl-[1,3,2]dioxaborolan-2-yl)-phenyl]-1*H*-pyrazole (**29a**)

Using the same boronylation procedure as described for **26,** from 3-(4-bromo-phenyl)-1-(4-methoxy-benzyl)*-1H-*pyrazole **28a** (350 mg, 1.0 mmol) the title compound was obtained (349 mg: 88%). ^1^H NMR (360 MHz, CDCl_3_) *δ* ppm: 7.83 (4H, s), 7.32 (1H, d, *J* = 2.7 Hz), 7.24 (2H, d, *J* = 8.6 Hz), 6.89 (2H, d, *J* = 8.6 Hz), 6.60 (1H, d, *J* = 2.7 Hz), 5.31 (2H, s), 3.81 (3H, s), 1.37 (12H, s). LCMS: *t*_R_ = 2.49 min, 391 (M+H)^+^ calcd for C_23_H_28_BN_2_O_3_. HRMS: (M)^+^ calcd for C_23_H_27_BN_2_O_3_: 390.2229, found: 390.2234.

##### 4.1.6.7 1-(4-Methoxy-benzyl)-4-methyl-3-[4-(4,4,5,5-tetramethyl-[1,3,2]dioxaborolan-2-yl)-phenyl]-1*H*-pyrazole (**29b**)

Standard boronylation procedure using 3-(4-bromo-phenyl)-1-(4-methoxy-benzyl)-4-methyl*-1H-*pyrazole **28b** (357 mg, 1.0 mmol) gave the desired compound (284 mg, 70%). ^1^H NMR (250 MHz, CDCl_3_) *δ* ppm: 7.86 (2H, d, *J* = 7.5 Hz), 7.72 (2H, d, *J* = 7.9 Hz), 7.24 (2H, d, *J* = 8.7 Hz), 7.14 (1H, s), 6.89 (2H, d, *J* = 8.7 Hz), 5.24 (2H, s), 3.81 (3H, s), 2.21 (3H, s), 1.37 (12H, s). HRMS: (M+H)^+^ calcd for C_24_H_29_BN_2_O_3_: 404.2386, found: 404.2390.

##### 4.1.6.8 3-(4-Bromo-phenyl)-4-chloro-1*H*-pyrazole (**30**)

3-(4-Bromophenyl)-pyrazole **27a** (223 mg, 1.0 mmol) and *N*-chlorosuccinimide (140 mg, 1.05 mmol) were dissolved in DCM (30 mL) and stirred at room temperature for 84 h. The solvent was evaporated and purified on a silica gel column with 10–25% EtOAc in heptane to yield the desired product **30**, 190 mg (74%). ^1^H NMR (360 MHz, CDCl_3_) *δ* ppm: 7.69 (2H, d, *J* = 5.8 Hz), 7.62 (1H, s), 7.58 (2H, d, *J* = 5.4 Hz). LCMS: *t*_R_ = 2.04 min, 257 (M+H)^+^ calcd for C_9_H_6_BrClN_2_.

##### 4.1.6.9 3-(4-Bromo-phenyl)-4-chloro-pyrazole-1-carboxylic acid *tert*-butyl ester (**31**)

4-Chloro-3-(4′-bromophenyl)-pyrazole **30** (190 mg, 0.74 mmol) was dissolved in acetonitrile (2.3 mL) with heating. After cooling on ice, BOC anhydride (193 mg, 0.89 mmol) and DMAP (9 mg; 0.1 mmol) were added and stirring continued at 0 °C for 2 h and room temperature for 1 h. The reaction was diluted with EtOAc (2 mL) and washed with 1.2 N HCl (2 × 2 mL), saturated sodium bicarbonate (2 mL) and brine (2 mL) before drying (Na_2_SO_4_), filtering and evaporating to leave the product as an oil. Yield = 244 mg (92%). ^1^H NMR (250 MHz, CDCl_3_) *δ* ppm: 8.11 (1H, s), 7.87 (2H, d, *J* = 8.7 Hz), 7.58 (2H, d, *J* = 8.8 Hz), 1.66 (9H, s). LCMS: *t*_R_ = 2.05 min, 298/300/302 (M+H-56 Boc fragmentation)^+^ calcd for C_14_H_14_BrClN_2_O_2_.

##### 4.1.6.10 4-Chloro-3-[4-(4,4,5,5-tetramethyl-[1,3,2]dioxaborolan-2-yl)-phenyl]-1*H*-pyrazole (**32**)

Using the same boronylation procedure as described for compound **26**, with 3-(4-bromo-phenyl)-4-chloro-pyrazole-1-carboxylic acid *tert*-butyl ester **31** (244 mg, 0.68 mmol) caused the loss of the BOC group. A 1:1 mix of unprotected 4-chloro-3-[4-(4,4,5,5-tetramethyl-[1,3,2]dioxa borolan-2-yl)-phenyl]*-1H-*pyrazole **32** and unprotected 4-chloro-3-(4′-bromophenyl)-pyrazole **30** was isolated after chromatography on silica gel (116 mg, 61%). ^1^H NMR (360 MHz, CDCl_3_) *δ* ppm: 7.91 (2H, d, *J* = 8.7 Hz), 7.79 (2H, d, *J* = 8.2 Hz), 7.60 (1H, s), 1.38 (12H, s). LCMS: *t*_R_ = 2.05 min, 257/259/261 (M+H)^+^ calcd for C_9_H_6_BrClN_2_ and 2.23 min, 305/307 (M+H)^+^ calcd for C_15_H_18_BClN_2_O_2_.

##### 4.1.6.11 4-Chloro-1-(4-methoxy-benzyl)-3-[4-(4,4,5,5-tetramethyl-[1,3,2]dioxaborolan-2-yl)-phenyl]-1*H*-pyrazole (**33**)

The (1:1) mixture of **30** and **32** resulted from the previous reaction (116 mg, 0.41 mmol) was dissolved in anhydrous DMF (1 mL) and added to 60% NaH in mineral oil (18 mg, 0.45 mmol) previously washed with heptane. After stirring for 10 min at room temperature, 4-methoxybenzyl chloride (0.06 mL, 0.45 mmol) was added and stirring continued at room temperature overnight. Water (2.5 mL) was then added and the oil extracted with EtOAc (2 × 2 mL). The combined organic phases were washed with saturated NaHCO_3_ (2 × 2 mL), dried (MgSO_4_), filtered and evaporated to leave a 2:1 mix of boronyl and bromophenyl PMB protected pyrazoles (112 mg, ∼50% Br; 75%). ^1^H NMR (360 MHz, CDCl_3_) *δ* ppm: 7.82 (2H, d, *J* = 8.6 Hz), 7.56 (2H, d, *J* = 8.6 Hz), 7.34 (1H, s), 7.25 (2H, dd, *J* = 8.6, 2.3 Hz), 6.91 (2H, d, *J* = 8.6 Hz), 5.22 (2H, s), 3.82 (3H, s), 1.37 (12H, s).

The crude mixture was subjected to standard boronylation procedure (as described for compound **26**) affording the desired compound (45 mg, 38%) after chromatography and crystallisation from heptane. ^1^H NMR (360 MHz, CDCl_3_) *δ* ppm: 7.85 (2H, d, *J* = 8.3 Hz), 7.79 (2H, d, *J* = 8.3 Hz), 7.26 (1H, s), 7.16 (2H, d, *J* = 8.5 Hz), 6.83 (2H, d, *J* = 8.6 Hz), 5.16 (2H, s), 3.74 (3H, s), 1.29 (12H, s). LCMS: *t*_R_ = 2.73 min, 425, 4 (M+H)^+^ calcd for C_23_H_29_BClN_2_O_3_.

#### 4.1.7 Synthesis and characterization of intermediates **34**–**43** (Scheme 6)

##### 4.1.7.1 2-(4-Bromo-benzoyl)-3-dimethylamino-acrylonitrile (**34**)

4-Bromophenyl-2 oxopropionitrile (448 mg, 2.0 mmol) was dissolved in DMF (2 mL) and tris(dimethylamino)methane (1.04 mL, 6.0 mmol) was added. The reaction was heated at 70 °C for 4 h before cooling on ice and adding water (7 mL). The desired product was filtered off, washed with water and dried. Yield = 339 mg (61%). ^1^H NMR (250 MHz, CDCl_3_) *δ* ppm: 7.97 (1H, s), 7.68 (2H, d, *J* = 10.2 Hz), 7.57 (2H, d, *J* = 9.9 Hz), 3.50 (3H, s), 3.32 (3H, s). The product was used in the next step without further purification.

##### 4.1.7.2 3-(4-Bromo-phenyl)-1*H*-pyrazole-4-carbonitrile (**35**)

2-(4-Bromo-benzoyl)-3-dimethylamino-acrylonitrile **34** (339 mg, 1.22 mmol) was suspended in ethanol (3.4 mL) and hydrazine hydrate (77 mL, 1.58 mmol) was added. The starting material dissolved and the reaction was stirred at room temperature for 3 h. The solvent was evaporated and water (5 mL) added. The solid was filtered off and chromatographed on silica gel with 60:40 EtOAc/heptane to give the desired product (233 mg; 77%). ^1^H NMR (250 MHz, acetone-*d_6_*): *δ* ppm: 8.44 (1H, s), 7.92 (2H, d, *J* = 8.5 Hz), 7.68–7.74 (3H, m). LCMS: *t*_R_ = 1.85 min, 248 (M+H)^+^ calcd for C_10_H_6_BrN_3_.

##### 4.1.7.3 3-(4-Bromo-phenyl)-1-(4-methoxy-benzyl)-1*H*-pyrazole-4-carbonitrile (**36**)

3-(4-Bromo-phenyl)*-1H-*pyrazole-4-carbonitrile **35** (233 mg, 0.94 mmol) was dissolved in anhydrous DMF (2.3 mL) and added to 60% NaH in mineral oil (41 mg, 1.03 mmol) previously washed with heptane. After stirring for 10 min at room temperature, 4-methoxybenzyl chloride (0.14 mL; 1.03 mmol) was added and stirring continued at room temperature for a further 4 h. Water (5 mL) was then added and an oil separated which crystallised with stirring. This was filtered and washed with water to leave the product (309 mg, 89%). ^1^H NMR (360 MHz, CDCl_3_) *δ* ppm: 7.87 (2H, d, *J* = 8.6 Hz), 7.72 (1H, s), 7.59 (2H, d, *J* = 8.6 Hz), 7.27 (2H, t, *J* = 4.3 Hz), 6.94 (2H, d, *J* = 8.6 Hz), 5.28 (2H, s), 3.83 (3H, s). The product was used in the next step without further purification.

##### 4.1.7.4 1-(4-Methoxy-benzyl)-3-[4-(4,4,5,5-tetramethyl-[1,3,2]dioxaborolan-2-yl)-phenyl]-1*H*-pyrazole-4-carbonitrile (**37**)

Standard boronylation procedure (as described for compound **26**) using 3-(4-bromo-phenyl)-1-(4-methoxy-benzyl)*-1H-*pyrazole-4-carbonitrile **36** (309 mg, 0.84 mmol) gave the desired compound (237 mg, 68%). ^1^H NMR (250 MHz, CDCl_3_) *δ* ppm: 7.99 (2H, d, *J* = 8.8 Hz), 7.90 (2H, d, *J* = 8.8 Hz), 7.72 (1H, s), 7.28 (2H, d, *J* = 8.7 Hz), 6.94 (2H, d, *J* = 8.7 Hz), 5.29 (2H, s), 3.83 (3H, s), 1.37 (12H, s). LCMS: *t*_R_ = 2.54 min, 416 (M+H)^+^ calcd for C_24_H_27_BN_3_O_3_. HRMS: (M)^+^ calcd for C_24_H_26_BN_3_O_3_: 415.2182, found: 415.2175.

##### 4.1.7.5 7-Bromo-4,5-dihydro-1*H*-benzo[*g*]indazole (**38**)

6-Bromo-3,4-dihydro*-2H-*naphthalen-1-one (204 mg, 0.91 mmol) was dissolved in DMF (2 mL) and tris-dimethylamino methane (657 mg, 4.53 mmol) was added. The reaction was stirred for 1 h, then further tris-dimethylamino methane (657 mg, 4.53 mmol) was added, and the reaction stirred overnight. The solvents were removed under vacuum and the residue azeothroped with heptane. The crude was then dissolved in AcOH (2 mL) and treated with NH_2_NH_2_·H_2_O (227 mg, 4.53 mmol). The reaction was stirred for 15 min, upon which concd NH_4_OH solution was added to pH 11. NaHCO_3_ saturated solution (10 mL) was added and the aqueous layer was extracted with DCM (3 × 10 mL), dried (MgSO_4_) and the solvent removed in vacuo to yield **38** as a red brick solid. Yield: 224 mg, 99%. ^1^H NMR (360 MHz, CDCl_3_) *δ* ppm: 7.89 (1H, d, *J* = 8.2 Hz), 7.73 (1H, s), 7.43 (1H, dd, *J* = 8.4, 2.0 Hz), 7.34 (1H, d, *J* = 1.8 Hz), 2.88–2.97(2H, m), 2.79–2.85 (2H, m). LCMS: *t*_R_ = 1.91 min, 249 (M+H)^+^ calcd for C_11_H_9_BrN_2_.

##### 4.1.7.6 7-Bromo-1-(4-methoxy-benzyl)-4,5-dihydro-1*H*-benzo[*g*]indazole (**39**)

NaH (60% in mineral oil, 15.6 mg, 0.39 mmol) was suspended in DMF (3.5 mL) and 7-bromo-4,5-dihydro*-1H-*benzo[*g*]indazole **38** (90 mg, 0.36 mmol) was added in DMF (1 mL). The suspension was stirred for 10 min and PMBCl (61 mg, 0.39 mmol) was added dropwise. The reaction was stirred at room temperature for 1 h. Water (10 mL) was added and the aqueous layer was extracted with EtOAc (3 × 10 mL). The organic layer was washed with water (2 × 10 mL) and brine (10 mL), dried (MgSO_4_), filtered, and the solvent removed under vacuum. The residue was purified by chromatography using 5% EtOAc in heptane to yield **39** as a yellow semi-solid. Yield: 62 mg, 47%. ^1^H NMR (360 MHz, CDCl_3_) *δ* ppm: 7.64 (1H, d, *J* = 8.2 Hz), 7.27–7.33 (2H, m), 7.15 (2H, d, *J* = 8.6 Hz), 7.01 (1H, s), 6.81 (2H, d, *J* = 8.6 Hz), 5.17 (2H, s), 3.72 (3H, s), 2.77–2.84 (2H, m), 2.60–2.67 (2H, m). LCMS: *t*_R_ = 2.50 min, 369 (M+H)^+^ calcd for C_19_H_17_BrN_2_O.

##### 4.1.7.7 1-(4-Methoxy-benzyl)-7-(4,4,5,5-tetramethyl-[1,3,2]dioxaborolan-2-yl)-4,5-dihydro-1*H*-benzo[*g*]indazole (**40**)

Standard boronylation procedure (as described for compound **26**) using intermediate **39** (62 mg, 0.17 mmol) resulted in the desired boronic acid **40** (contaminated with 30% of the reduced material, 70% corrected yield). This mixture was used in the next step without further purification. ^1^H NMR (250 MHz, CDCl_3_) *δ* ppm: 7.82–7.90 (1H, m), 7.65–7.76 (2H, m), 7.24 (2H, d, *J* = 8.8 Hz), 7.08 (1H, s), 6.89 (2H, d, *J* = 8.7 Hz), 5.27 (2H, s), 3.81 (5H, s), 2.93 (2H, t, *J* = 7.1 Hz), 2.72 (2H, t, *J* = 7.2 Hz), 1.36 (12H, s). LCMS: *t*_R_ = 2.62 min, 417 (M+H)^+^ calcd for C_25_H_29_BN_2_O.

##### 4.1.7.8 7-Bromo-1*H*-benzo[*g*]indazole (**41**)

Compound **38** (170 mg, 0.68 mmol) was dissolved in dioxane (6 mL) and DDQ (310 mg, 1.36 mmol) was added. The solution was refluxed for 3 h and the solvent was removed under vacuum. 2 M NaOH (10 mL) was added, and the aqueous layer was extracted with TBME (3 × 10 mL), dried (MgSO_4_) and the solvent removed under vacuum. The residue was purified by chromatography using a stepped gradient of 20–40% EtOAc in heptane to yield **41** as red brick solid. Yield: 81 mg, 48%. ^1^H NMR (250 MHz, CDCl_3_) *δ* ppm: 8.10–8.18 (2H, m), 8.07 (1H, d, *J* = 8.5 Hz), 7.67–7.78 (2H, m), 7.44 (1H, d, *J* = 8.5 Hz). LCMS: *t*_R_ = 1.96 min, 247 (M+H)^+^ calcd for C_11_H_7_BrN_2_.

##### 4.1.7.9 7-Bromo-1-(4-methoxy-benzyl)-1*H*-benzo[*g*]indazole (**42**)

NaH (60% in mineral oil, 8.6 mg, 0.36 mmol) was suspended in DMF (2 mL) and **41** (81 mg, 0.33 mmol) was added in DMF (1 mL). The suspension was stirred for 10 min and PMBCl (56 mg, 0.36 mmol) was added dropwise. The reaction was stirred at room temperature for 1 h. Water (10 mL) was added and the aqueous layer was extracted with TBME (3 × 10 mL). The organic layer was washed with water (2 × 10 mL) and brine (10 mL), dried (MgSO_4_), filtered, and the solvent removed under vacuum. The residue was purified by chromatography using 10% EtOAc in heptane to yield **42** as a yellow solid. Yield: 93 mg, 77%. ^1^H NMR (250 MHz, CDCl_3_) *δ* ppm: 8.47 (1H, d, *J* = 8.7 Hz), 7.96 (1H, d, *J* = 1.8 Hz), 7.81 (1H, s), 7.67 (1H, dd, *J* = 8.5, 2.0 Hz), 7.51 (1H, d, *J* = 9.0 Hz), 7.31 (1H, s), 6.91 (2H, d, *J* = 8.7 Hz), 5.57 (2H, s), 3.81 (3H, s). LCMS: *t*_R_ = 2.47 min, 367 (M+H)^+^ calcd for C_19_H_15_BrN_2_O.

##### 4.1.7.10 1-(4-Methoxy-benzyl)-7-(4,4,5,5-tetramethyl-[1,3,2]dioxaborolan-2-yl)-1*H*-benzo[*g*]indazole (**43**)

Standard boronylation procedure (as described for compound **26**) using intermediate **42** (93 mg, 0.25 mmol) resulted in the desired boronic acid **43** (53 mg, 51%). ^1^H NMR (250 MHz, CDCl_3_) *δ* ppm: 8.59 (1H, d, *J* = 8.1 Hz), 8.31 (1H, s), 7.99 (1H, d, *J* = 6.9 Hz), 7.79 (1H, s), 7.36–7.51 (2H, m), 7.31(2H, d), 6.91 (2H, d, *J* = 8.7 Hz), 5.59 (2H, s), 3.81 (3H, s), 1.41 (12H, s). LCMS: *t*_R_ = 2.59 min, 415 (M+H)^+^ calcd for C_25_H_27_BN_2_O.

#### 4.1.8 Synthesis of final compounds **1a**–**1p**

##### 4.1.8.1 Dimethyl-[2-(4-{5-[4-(1*H*-pyrazol-3-yl)-phenyl]-4-pyridin-4-yl-1*H*-imidazol-2-yl}-phenoxy)-ethyl]-amine (**1a**)

{2-[4-(5-{4-[1-(4-Methoxy-benzyl)-*1H*-pyrazol-3-yl]-phenyl}-1-methoxymethyl-4-pyridin-4-yl-*1H*-imidazol-2-yl)-phenoxy]-ethyl}-dimethyl-amine **8a** (47 mg; 0.077 mmol) and anisole (17 μL; 0.153 mmol) were dissolved in TFA (1.5 mL) and heated at 130 °C for 4 h then cooled to room temperature overnight. The solvent was evaporated, the residue washed with TBME and purified by preparative LC to give the desired compound (11 mg; 32%). ^1^H NMR (360 MHz, MeOH) *δ* ppm: 8.65 (2H, d, *J* = 5.4 Hz), 8.20 (2H, d, *J* = 6.4 Hz), 8.12 (2H, d, *J* = 9.1 Hz), 8.05 (2H, d, *J* = 8.2 Hz), 7.80 (1H, s), 7.72 (2H, d, *J* = 8.2 Hz), 7.26 (2H, d, *J* = 9.1 Hz), 6.85 (1H, s), 4.47–4.54 (2H, m), 3.67–3.73 (2H, m), 3.06 (6H, s). LCMS: *t*_R_ = 2.36 min, 451 (M+H)^+^ calcd for C_27_H_26_N_6_O. HRMS: (M+H)^+^ calcd for C_27_H_26_N_6_O: 451.2246, found: 451.2235.

##### 4.1.8.2 Dimethyl-[2-(4-{5-[4-(4-methyl-1*H*-pyrazol-3-yl)-phenyl]-4-pyridin-4-yl-1*H*-imidazol-2-yl}-phenoxy)-ethyl]-amine (**1b**)

{2-[4-(5-{4-[1-(4-Methoxy-benzyl)-4-methyl-*1H*-pyrazol-3-yl]-phenyl}-1-methoxy methyl-4-pyridin-4-yl-*1H*-imidazol-2-yl)-phenoxy]-ethyl}-dimethyl-amine **8b** (60 mg; 0.096 mmol) and anisole (21 μl; 0.192 mmol) were dissolved in TFA (1.5 mL) and heated at 130 °C for 4 h then cooled to room temperature overnight. The solvent was evaporated, the residue washed with TBME and purified by preparative LC to give the desired compound (8 mg; 18%). ^1^H NMR (250 MHz, acetone) *δ* ppm: 11.80 (1H, br s), 8.56 (1H, d, *J* = 5.2 Hz), 8.46 (1H, d, *J* = 6.2 Hz), 8.08 (2H, d, *J* = 8.8 Hz), 7.83 (1H, d, *J* = 8.4 Hz), 7.73 (1H, s), 7.63–7.66 (2H, m), 7.61 (1H, s), 7.56 (1H, s), 7.52 (1H, d, *J* = 5.9 Hz), 7.06 (2H, d, *J* = 9.0 Hz), 4.16 (2H, t, *J* = 5.9 Hz), 2.70 (2H, t, *J* = 5.9 Hz), 2.30 (3H, s), 2.28 (6H, s). LCMS: *t*_R_ = 2.45 min, 465 (M+H)^+^ calcd for C_28_H_29_N_6_O. HRMS: (M+H)^+^ calcd for C_28_H_29_N_6_O: 465.2403, found: 465.2400.

##### 4.1.8.3 3-(4-{2-[4-(2-Dimethylamino-ethoxy)-phenyl]-5-pyridin-4-yl-3*H*-imidazol-4-yl}-phenyl)-1*H*-pyrazole-4-carbonitrile (**1c**)

3-(4-{2-[4-(2-Dimethylamino-ethoxy)-phenyl]-3-methoxymethyl-5-pyridin-4-yl-*3H*-imidazol-4-yl}-phenyl)-1-(4-methoxy-benzyl)-*1H*-pyrazole-4-carbonitrile **8c** (61 mg; 0.096 mmol) was dissolved in TFA (1.2 mL) and heated at 70 °C for 4 h then cooled to room temperature overnight. The solvent was evaporated, the residue washed with TBME and purified by preparative LC to give the desired compound (10 mg; 22%). ^1^H NMR (250 MHz, acetone) *δ* ppm: 8.51 (2H, dd, *J* = 4.6, 1.5 Hz), 8.43 (1H, s), 8.07 (4H, dd), 7.75 (2H, d, *J* = 8.5 Hz), 7.61 (2H, dd, *J* = 4.6, 1.5 Hz), 7.06 (2H, d, *J* = 9.0 Hz), 4.16 (2H, t, *J* = 5.9 Hz), 2.70 (2H, t, *J* = 5.9 Hz), 2.28 (6H, s). LCMS: *t*_R_ = 2.42 min, 476 (M+H)^+^ calcd for C_28_H_25_N_7_O. HRMS: (M+H)^+^ calcd for C_28_H_25_N_7_O: 476.2199, found: 476.2194.

##### 4.1.8.4 [2-(4-{5-[4-(4-Chloro-1*H*-pyrazol-3-yl)-phenyl]-4-pyridin-4-yl-1*H*-imidazol-2-yl}-phenoxy)-ethyl]-dimethyl-amine (**1d**)

{2-[4-(5-{4-[4-Chloro-1-(4-methoxy-benzyl)-*1H*-pyrazol-3-yl]-phenyl}-4-pyridin-4-yl-*1H*-imidazol-2-yl)-phenoxy]-ethyl}-dimethyl-amine **9d** (18 mg; 0.03 mmol) was dissolved in TFA (0.5 mL) and heated at 65 °C for 11 h then cooled to room temperature overnight. The solvent was evaporated, the residue washed with TBME to give the desired compound (19 mg; 76%). ^1^H NMR (360 MHz, MeOH/ CDCl_3_) *δ* ppm: 8.54 (2H, br s), 8.11 (2H, d, *J* = 5.4 Hz), 8.04 (4 H, dd, *J* = 8.6, 2.7 Hz), 7.72 (1H, s), 7.66 (2H, d, *J* = 8.2 Hz), 7.14 (2H, d, *J* = 9.1 Hz), 4.42 (2H, t), 3.61 (2H, t), 2.99 (6H, s). LCMS: *t*_R_ = 2.55 min, 485 (M+H)^+^ calcd for C_27_H_25_ClN_6_O. HRMS: (M+H)^+^ calcd for C_27_H_25_ClN_6_O: 485.1856, found: 485.1873.

##### 4.1.8.5 (2-{4-[5-(4,5-Dihydro-2*H*-benzo[*g*]indazol-7-yl)-4-pyridin-4-yl-1*H*-imidazol-2-yl]-phenoxy}-ethyl)-dimethyl-amine (**1e**)

[2-(4-{4-[1-(4-Methoxy-benzyl)-4,5-dihydro-*1H*-benzo[*g*]indazol-7-yl]-1-methoxy methyl-5-pyridin-4-yl-*1H*-imidazol-2-yl}-phenoxy)-ethyl]-dimethyl-amine **8e** (13 mg; 0.02 mmol) was dissolved in TFA (1.5 mL), and anisole (4.4 mg; 0.04 mmol) was added. The solution was heated in a sealed vessel for 24 h. The solvent was removed under vacuum and the residue purified by preparative HPLC to yield the desired compound **1e**. TFA salt as a yellow glue (1 mg, 6%). ^1^H NMR (360 MHz, MeOD) *δ* ppm: 8.58 (2H, d, *J* = 6.4 Hz), 8.16 (2H, d, *J* = 6.8 Hz), 8.07 (2H, d, *J* = 9.1 Hz), 7.91 (1H, d, *J* = 8.2 Hz), 7.54 (2H, d, *J* = 5.0 Hz), 7.50 (1H, d, *J* = 7.3 Hz), 7.21 (2H, d, *J* = 9.1 Hz), 4.44–4.48 (2H, m), 3.63–3.68 (2H, m), 3.49–3.52 (1H, m), 3.10–3.13 (1H, m), 3.02 (6H, s), 2.83–2.89 (2H, m). LCMS: *t*_R_ = 2.50 min, 477 (M+H)^+^ calcd for C_29_H_29_N_6_O. HRMS: (M+H)^+^ calcd for C_29_H_29_N_6_O: 477.2403, found: 477.2400.

##### 4.1.8.6 (2-{4-[4-(1*H*-Benzo[*g*]indazol-7-yl)-5-pyridin-4-yl-1*H*-imidazol-2-yl]-phenoxy}-ethyl)-dimethyl-amine (**1f**)

Using the same procedure as for **1e**, **1f** was obtained from **8f** (33 mg; crude). Yield: 5 mg (8% over two steps). ^1^H NMR (250 MHz, MeOD) *δ* ppm: 8.52–8.62 (3H, m), 8.27 (1H, d, *J* = 1.37 Hz), 8.22 (1H, s), 8.07–8.16 (4H, m), 7.79–7.89 (2H, m), 7.60 (1H, d, *J* = 8.98 Hz), 7.23 (2H, d, *J* = 8.98 Hz), 4.47 (2H, t), 3.66 (2H, t), 3.02 (s, 6H). LCMS: *t*_R_ = 2.33 min, 475 (M+H)^+^ calcd for C_29_H_27_N_6_O. HRMS: (M+H)^+^ calcd for C_29_H_27_N_6_O: 475.2246, found: 475.2244.

##### 4.1.8.7 7-{2-[4-(2-Dimethylamino-ethoxy)-phenyl]-5-pyridin-4-yl-1*H*-imidazol-4-yl}-2,4,4a,5-tetrahydro-indeno[1,2-*c*]pyridazin-3-one (**1g**)

Using the same procedure as for **1e**, **1g** (2.4 mg, 47%) was obtained from **8g**. ^1^H NMR (360 MHz, MeOD) *δ* ppm: 8.57 (2H, br s), 8.05 (2H, d, *J* = 8.9 Hz), 8.01 (2H, br s), 7.85 (1H, d, *J* = 7.9 Hz), 7.66 (1H, s), 7.56 (1H, d, *J* = 7.9 Hz), 7.20 (2H, d, *J* = 8.9 Hz), 4.41–4.50 (2H, m), 3.61–3.69 (2H, m), 3.44–3.55 (1H, m), 3.01 (6H, s), 2.79–2.94 (2H, m), 2.45 (1H, t, *J* = 16.3 Hz). LCMS: *t*_R_ = 2.19 min, 493 (M+H)^+^ calcd for C_29_H_29_N_6_O_2_. HRMS: (M+H)^+^ calcd for C_29_H_29_N_6_O_2_: 493.2352, found: 493.2347.

##### 4.1.8.8 6-{2-[4-(4-Methyl-piperazin-1-yl)-phenyl]-5-pyridin-4-yl-3*H*-imidazol-4-yl}-2,4-dihydro-indeno[1,2-*c*]pyrazole (**1j**)

Ketone **16a** (37.1 mg; 0.083 mmol) was dissolved in dry DMF (0.5 mL) and tris(dimethylamine)methane (86 μL; 0.50 mmol) was added, reaction was heated to 85 °C, under N_2_ for 3 h. Solvents were removed under vacuum and residue was dissolved in AcOH (0.5 mL). Hydrazine hydrate (12 μL; 0.25 mmol) was added to the reaction mixture and was then stirred overnight. Solvent was removed under vacuum and residue was purified by Flash column chromatography on silica gel, eluting the desired product in 10–50% EtOH/dichloromethane (DCM) (6 mg; 15%). ^1^H NMR (250 MHz, MeOD) *δ* ppm: 8.35 (2H, d, *J* = 8.1 Hz), 7.88 (2H, d, *J* = 8.8 Hz), 7.70 (1H, d, *J* = 7.9 Hz), 7.59 (2H, s), 7.51 (1H, s), 7.43 (1H, d, *J* = 7.6 Hz), 7.07 (2H, d, *J* = 9.0 Hz), 3.25–3.67 (10H, m), 2.87 (3H, s). LCMS: *t*_R_ = 1.11 min 474 (M+H)^+^ calcd for C_29_H_28_N_7_. HRMS: (M+H)^+^ calcd for C_29_H_28_N_7_: 474.2406, found: 474.2399.

##### 4.1.8.9 (2-{4-[5-(2,4-Dihydro-indeno[1,2-*c*]pyrazol-6-yl)-4-pyridin-4-yl-1*H*-imidazol-2-yl]-phenoxy}-ethyl)-dimethyl-amine (**1h**)

Using the same procedure as for **1j** from **16b** (38 mg; 0.087 mmol) the desired **1h** was obtained (26 mg; 65%). ^1^H NMR (250 MHz, MeOD) *δ* ppm: 8.32 (2H, br s), 7.84 (2H, d, *J* = 8.7 Hz), 7.33–7.71 (6H, m), 6.97 (2H, d, *J* = 8.8 Hz), 4.06 (2H, t, *J* = 5.4 Hz), 3.58 (2H, s), 2.70 (2H, br s), 2.26 (6H, s). LCMS: *t*_R_ = 1.10 min, 463 (M+H)^+^ calcd for C_28_H_27_N_6_O. HRMS: (M+H)^+^ calcd for C_28_H_27_N_6_O: 463.2246, found: 463.2242.

##### 4.1.8.10 6-(2-Piperidin-4-yl-5-pyridin-4-yl-3*H*-imidazol-4-yl)-2,4-dihydro-indeno[1,2-*c*]pyrazole (**1k**)

Using the same procedure as for **1j** from **16c** (33 mg; 0.069 mmol), the *tert*-butoxycarbonyl (Boc)-protected version of **1k** was obtained. The residue was dissolved in DCM (5 mL) and 4 N HCl (0.09 mL) added. After stirring at rt for 4 h, the solvents were evaporated under vacuum and the residue triturated with diethyl ether to leave the desired product as the HCl salt of **1k** (33 mg; 100%). ^1^H NMR (250 MHz, MeOD) *δ* ppm: 8.83 (2H, br s), 8.20 (2H, br s), 8.07 (1H, s), 7.64–8.02 (3H, m), 3.94 (2H, br s), 3.63 (4H, d, *J* = 14.3 Hz), 2.20–2.57 (5H, m). LCMS: *t*_R_ = 0.93 min, 383 (M+H)^+^ calcd for C_23_H_22_N_6_. HRMS: (M+H)^+^ calcd for C_23_H_22_N_6_: 383.1984, found: 383.1077.

##### 4.1.8.11 (2-{4-[5-(3,8-Dihydro-indeno[1,2-*d*] [1,2,3]triazol-6-yl)-4-pyridin-4-yl-1*H*-imidazol-2-yl]-phenoxy}-ethyl)-dimethyl-amine (**1i**)

To a solution of ketone **16b** (55 mg; 0.144 mmol) in 1 mL of methoxyethanol/HCl concd (3:1) at 0 °C was added *t*BuONO (30 μL; 0.29 mmol). The reaction was stirred at 0 °C for 1 min then more *t*BuONO (30 μL; 0.29 mmol) were added. The solution was stirred for 30 min at 0 °C. Slowly 2 mL of NaOH (2 M) were added. The solution was extracted with EtOAc (5 mL). The solvent was removed under vacuo and the oily residue was dissolved in 8 mL of ethylene glycol/KOH (85%) (10:1). Hydrazine hydrate (800 mg; 16 mmol) was added and the solution was heated at 190 °C for 2 h in a boiling tube. The reaction was allowed to cool to room temperature, diluted with 5 mL of water and extracted with DCM (10 mL). The aqueous layer was evaporated under vacuum. The residue was loaded on Ambersep (sulfonic acid) resin (10 mL). The resin was washed with MeOH (3 × 10 mL), H_2_O (10 mL), HCl (1 M, 5 × 10 mL) then cleaved with HCl (5 M, 5 × 10 mL). The fractions were evaporated under vacuum. The residue was suspended in 10 mL IPA/EtOH (1:1), filtered. The solvent was removed under vacuum, the residue was purified by reverse phase column chromatography (C18) eluting with MeOH to afford 1.9 mg of **1i** (3%). ^1^H NMR (360 MHz, MeOD) *δ* ppm: 8.56 (2H, br s), 8.03–8.10 (3H, m), 7.92 (1H, d, *J* = 7.7 Hz), 7.84 (1H, s), 7.65 (1H, d, *J* = 7.7 Hz), 7.21 (2H, d, *J* = 9.1 Hz), 4.46 (2H, t, *J* = 10.0 Hz), 3.91 (2H, s), 3.66 (2H, t, *J* = 10.0 Hz), 3.02 (6H, s) LCMS: *t*_R_ = 2.30 min, 464 (M+H)^+^ calcd for C_27_H_26_N_7_O. HRMS: (M+H)^+^ calcd for C_27_H_26_N_7_O: 464.2199, found: 464.2193.

##### 4.1.8.12 [4-(2,4-Dihydro-indeno[1,2-*c*]pyrazol-6-yl)-5-pyridin-4-yl-furan-2-yl]-[4-(2-dimethylamino-ethyl)-piperazin-1-yl]-methanone (**1l**)

Ketone **22b** (86 mg; 0.19 mmol) was dissolved in dry DMF (1 mL) and DMF–DMA (112 mg; 0.94 mmol) was added. The reaction was heated to 85 °C for 3 h. Solvent was removed under vacuum and remaining DMF was azeothroped with EtOH/heptane. The residue was dissolved in AcOH (1 mL) and the hydrazine hydrate (45 μL; 0.94 mmol) was added. Reaction was stirred at room temperature overnight. The reaction mixture was basified to pH 11 with NH_4_OH (concd), extracted with EtOAc (4 × 6 mL), dried (MgSO_4_) filtered and evaporated to dryness to leave a yellow solid. This solid was dissolved in 1 M HCl (10 mL) and washed with EtOAc (2 × 5 mL). Aqueous was basified with 2 M NaOH to pH 11 and extracted again with EtOAc, dried (MgSO_4_), filtered and evaporated to dryness to leave a cream solid. Yield: 20 mg (22%). ^1^H NMR (360 MHz, MeOH) *δ* ppm: 8.49 (2H, d, *J* = 7.5 Hz), 7.79 (1H, d, *J* = 7.8 Hz), 7.55–7.66 (4H, m), 7.45 (1H, d, *J* = 7.8 Hz), 7.24 (1H, s), 3.94 (4H, br s), 3.70 (2H, s), 2.53–2.68 (8H, m), 2.32 (6H, s). LCMS: *t*_R_ = 1.16 min, 483 (M+H)^+^ calcd for C_28_H_30_N_6_O_2_. HRMS: (M+H)^+^ calcd for C_28_H_30_N_6_O_2_: 483.2508, found: 483.2514.

##### 4.1.8.13 [4-(2,4-Dihydro-indeno[1,2-*c*]pyrazol-6-yl)-5-pyridin-4-yl-furan-2-yl]-morpholin-4-yl-methanone (**1m**)

Starting from ketone **22c** (96 mg; 0.25 mmol) and using the same procedure as for compound **1l** the desired compound was obtained (18 mg; 11%). ^1^H NMR (250 MHz, MeOD) *δ* ppm: 8.48 (2H, br s), 7.79 (2H, d, *J* = 7.8 Hz), 7.54–7.63 (4H, m), 7.44 (1H, dd, *J* = 7.9, 1.4 Hz), 7.26 (1H, s), 3.91 (4H, br s), 3.75–3.84 (4H, m), 3.71 (2H, s). LCMS: *t*_R_ = 3.03 min, 413 (M+H)^+^ calcd for C_24_H_21_N_4_O_3._ HRMS: (M+H)^+^ calcd for C_24_H_21_N_4_O_3_: 413.1614, found: 413.1611.

##### 4.1.8.14 4-[4-(2,4-Dihydro-indeno[1,2-*c*]pyrazol-6-yl)-5-pyridin-4-yl-furan-2-carbonyl]-piperazine-1-carboxylic acid *tert*-butyl ester (**23d**)

Starting from ketone **22d** (106 mg; 0.22 mmol) and using the same procedure as for compound **1l** the desired compound was obtained (70 mg; 63%). ^1^H NMR (250 MHz, CDCl_3_-*d*) *δ* ppm: 8.53 (2H, br s), 7.81 (1H, d, *J* = 7.8 Hz), 7.45–7.57 (2H, m), 7.32–7.45 (3H, m), 7.18 (1H, s), 3.87 (4H, br s), 3.65–3.72 (2H, m), 3.49–3.64 (4H, m), 1.48 (9H, s). LCMS: *t*_R_ = 3.62 min, (M+H)^+^ calcd for C_29_H_29_N_5_O_4._

##### 4.1.8.15 [4-(2,4-Dihydro-indeno[1,2-*c*]pyrazol-6-yl)-5-pyridin-4-yl-furan-2-yl]-piperazin-1-yl-methanone (**1n**)

BOC amide **23d** was dissolved in DCM (0.5 mL) and dioxane (0.3 ml) and 4 N HCl in dioxane (0.3 mL) was added. After stirring at room temperature for 2 h, the solvents were evaporated under vacuum to leave the desired **1n** (30 mg; 100%). ^1^H NMR (250 MHz, MeOD) *δ* ppm: 8.74 (2H, d, *J* = 6.1 Hz), 8.16 (2H, d, *J* = 6.3 Hz), 8.08 (1H, s), 7.96 (1H, d, *J* = 7.8 Hz), 7.87 (1H, s), 7.68 (1H, d, *J* = 7.9 Hz), 7.47 (1H, s), 4.00–4.37 (4H, m), 3.86–3.98 (2H, m), 3.36–3.52 (4H, m). LCMS: *t*_R_ = 2.45 min, 412 (M+H)^+^ calcd for C_24_H_22_N_5_O_2_. HRMS: (M+H)^+^ calcd for C_24_H_22_N_5_O_2_: 412.1774, found: 412.1769.

##### 4.1.8.16 [4-(2,4-Dihydro-indeno[1,2-*c*]pyrazol-6-yl)-5-pyridin-4-yl-furan-2-yl]-(4-methyl-piperazin-1-yl)-methanone (**1o**)

Starting from ketone **22a** (79 mg; 0.20 mmol) and using the same procedure the desired compound was obtained (50 mg; 60%). ^1^H NMR (250 MHz, MeOD) *δ* ppm: 8.47 (2H, br s), 7.77 (1H, d, *J* = 7.8 Hz), 7.48–7.67 (4H, m), 7.43 (1H, d, *J* = 7.6 Hz), 7.22 (1H, s), 3.73–4.11 (4H, m), 3.68 (2H, s), 2.45–2.70 (4H, m), 2.36 (3H, s). LCMS: *t*_R_ = 2.41 min, 426 (M+H)^+^ calcd for C_25_H_23_N_5_O_2_. HRMS: (M+H)^+^ calcd for C_25_H_23_N_5_O_2_: 426.1930, found: 426.1934.

##### 4.1.8.17 [4-(2-Dimethylamino-ethyl)-piperazin-1-yl]-[4-(1-methyl-1,4-dihydro-indeno [1,2-*c*]pyrazol-6-yl)-5-pyridin-4-yl-furan-2-yl]-methanone (**1p**)

The starting ketone **22b** (34 mg, 0.07 mmol) was dissolved in dry DMF (0.5 mL) and DMF·DMA (44 mg; 0.37 mmol) was added. The reaction was stirred for 90 min. The solvents were removed under vacuum and the residue azeotroped with heptane. The crude was then dissolved in AcOH (0.5 mL) and treated with methylhydrazine (51 mg; 1.11 mmol). The reaction was stirred at room temperature for two days, upon which concentrated NH_4_OH solution was added to pH 11. NaHCO_3_ saturated solution (10 mL) was added and the aqueous layer was extracted with EtOAc (3 × 10 mL), dried (MgSO_4_) and the solvent removed under vacuum. The residue was purified by chromatography using a stepped gradient of 0–15% MeOH in DCM. The product was re-suspended in EtOAc (2 mL) and washed with water (5 × 1 mL). The organic layer was dried (MgSO_4_) and the solvent removed in vacuo to yield a pale yellow glue (3 mg; 8%). ^1^H NMR (250 MHz, MeOD) *δ* ppm: 8.46–8.52 (2H, m), 7.80 (1H, d), 7.64 (1H, s), 7.54–7.60 (2H, m), 7.45–7.52 (1H, m), 7.43 (1H, s), 7.25 (1H, s), 4.15 (3H, s), 3.91 (4H, br s), 3.64 (2H, s), 2.58–2.67 (8H, m), 2.34 (6H, s). LCMS: *t*_R_ = 2.73 min, 497 (M+H)^+^ calcd for C_29_H_33_N_6_O_2_. HRMS: (M+H)^+^ calcd for C_29_H_33_N_6_O_2_: 497.2665, found: 497.2660.

### 4.2 Docking and modelling

Inhibitor **1a** was docked using GOLD version 3.1.1[5] on the crystal structure of BRAF in complex with SB590885 [PDB 2FB8]. Partial charges of the ligand were derived using the Charge-2 CORINA 3D package in TSAR 3.3, and its geometry optimized using the COSMIC module of TSAR. The calculations were terminated if the energy difference or the energy gradient were smaller than 1E-005. Ten docking solutions were generated, and the best three stored for analysis.

### 4.3 Biology

#### 4.3.1 ^V600E^BRAF kinase assay and SRB IC_50_ for BRAF inhibitors

These assays have been described by Niculescu-Duvaz et al.^15^

#### 4.3.2 Phospho-ERK IC_50_ assay

To determine the effect of compounds on BRAF activity in cells, WM266.4 cells were seeded at a density of 3 × 10^4^ cells per well of a 96 well plate. The following day, test compounds were diluted into growth medium to 2× the desired final concentration and then added directly to the cells. After a 6 h incubation, the medium was removed and cells were fixed and permeabilized in 4% formaldehyde, 0.1% triton X-100 in PBS for 30 min. The wells were then blocked with 5% milk in PBS for 30 min at room temperature, followed by the addition of an antibody for phospho-ERK1/2 (Sigma, Dorset, UK) at 3 mg/ml in blocking solution. Plates were incubated for 3 h with shaking. Plates were washed three times with 0.1% Tween 20 using an ELx50 plate washer (BioTek, Winooski, USA). 0.5 mg/ml of a Europium-labelled anti-mouse secondary antibody (Perkin Elmer, Turku, Finland) was added to the wells in DELFIA assay buffer for 1 h. Plates were washed again and Enhancement solution was added to the wells and time resolved fluorescence was measured as instructed by the manufacturer after 20 min using a Spectramax M5 plate reader (Molecular Devices, Berkshire, UK). The plates were washed again and BCA protein assay reagent (Sigma, Dorset, UK) was added to the wells and incubated for 30 min at 37 °C. Absorbance at 570 nm was measured using a plate reader and used to normalize the fluorescence data. Inhibition of ERK phosphorylation was determined as a percentage of DMSO-treated cells and IC_50_ values were calculated using Prism (GraphPad Software, San Diego, USA).

#### 4.3.3 PK assessment

Female Crl:CD1-Foxn1nu mice at least six weeks of age bearing mutant BRAF WM266.4 human tumor xenografts were used for the PK analyses. The mice were dosed intraperitoneally (4 mg/kg, equivalent to ca. 8 μmol/kg, 10 ml/kg, in 10% ethanol:40% PEG:50% Gelofusine v/v) in a cassette with 4 other compounds. Samples were taken at 8 time-points between 15 min and 24 h. Three mice were utilized per time-point. They were placed under isoflurane anaesthesia and blood for plasma preparation was taken by terminal cardiac puncture into heparinized syringes. Plasma samples were snap frozen in liquid nitrogen and then stored at −70 °C prior to analysis. All procedures involving animals were performed in accordance with national Home Office regulations under the Animals (Scientific Procedures) Act 1986 and within guidelines set out by the Institute’s Animal Ethics Committee and the United Kingdom Coordinating Committee for Cancer Research’s ad hoc Committee on the Welfare of Animals in Experimental Neoplasia.

## 5 Disclosure statement

This work was carried out as part of a research collaboration between the Institute of Cancer Research, The Wellcome Trust, GSK and Cancer Research UK. Please note that all authors who are, or have been, employed by The Institute of Cancer Research are subject to a ‘Rewards to Inventors Scheme’ that may reward contributors to a programme that is subsequently licensed.

Andrew K. Takle and David M. Wilson were employees of GlaxoSmithKline at the time of the aforementioned research collaboration.

## Figures and Tables

**Table 1:** Biological activities of BRAF inhibitors

No	Compound	IC_50_ BRAF (μM)	IC_50_ ppERK (μM)	GI_50_ SRB (μM)
**1a**		1.60	5.8	7.4
**1b**		1.20	2.02	3.9
**1c**		>10	>100	11
**1d**		3.7	10	4.1
**1e**		1.84	5.8	—
**1f**		1.71	25	3.3
**1g**		>10	100	8.9
**1h**		0.23	1.09	1.15
**1i**		>10	4.4	—
**1j**		0.24	0.58	0.87
**1k**		1.33	4.9	36
**1l**		3.6	0.74	1.61
**1m**		2.15	0.84	0.94
**1n**		2.78	0.97	1.59
**1o**		7.5	0.73	1.91
**1p**		>10	>10	—

## MOL file

The following ZIP file contains the MOL file of the most important compounds referred to in this article.

MOL file ZIP file containing the MOL file of the most important compounds in this article.
